# *Nocardiopsis synnemataformans* NBRM9, an extremophilic actinomycete producing extremozyme cellulase, using lignocellulosic agro-wastes and its biotechnological applications

**DOI:** 10.3934/microbiol.2024010

**Published:** 2024-03-12

**Authors:** Mohamed H. El-Sayed, Doaa A. Elsayed, Abd El-Rahman F. Gomaa

**Affiliations:** 1 Department of Biology, College of Science and Arts, Northern Border University, Arar, Saudi Arabia; 2 Department of Botany and Microbiology, Faculty of Science, Al-Azhar University, Cairo 11884, Egypt; 3 Department of Botany and Microbiology, Faculty of Science, Al-Azhar University, Assiut Branch, Assiut 71524, Egypt; 4 The Key Laboratory of Biotechnology for Medicinal Plants of Jiangsu Province, School of Life Science, Jiangsu Normal University, Xuzhou, Jiangsu, 221116, PR China

**Keywords:** Cellulase, bean straw, statistical optimization, anti-biofilm, bioethanol

## Abstract

Actinomycetes are an attractive source of lignocellulose-degrading enzymes. The search for actinomycetes producing extremozyme cellulase using cheap lignocellulosic waste remains a priority goal of enzyme research. In this context, the extremophilic actinomycete NBRM9 showed promising cellulolytic activity in solid and liquid assays. This actinomycete was identified as *Nocardiopsis synnemataformans* based on its phenotypic characteristics alongside phylogenetic analyses of 16S rRNA gene sequencing (OQ380604.1). Using bean straw as the best agro-waste, the production of cellulase from this strain was statistically optimized using a response surface methodology, with the maximum activity (13.20 U/mL) achieved at an incubation temperature of 40 °C, a pH of 9, an incubation time of 7 days, and a 2% substrate concentration. The partially purified cellulase (PPC) showed promising activity and stability over a wide range of temperatures (20–90 °C), pH values (3–11), and NaCl concentrations (1–19%), with optimal activity at 50 °C, pH 9.0, and 10% salinity. Under these conditions, the enzyme retained >95% of its activity, thus indicating its extremozyme nature. The kinetics of cellulase showed that it has a V_max_ of 20.19 ± 1.88 U/mL and a Km of 0.25 ± 0.07 mM. The immobilized PPC had a relative activity of 69.58 ± 0.13%. In the in vitro microtiter assay, the PPC was found to have a concentration-dependent anti-biofilm activity (up to 85.15 ± 1.60%). Additionally, the fermentative conversion of the hydrolyzed bean straw by *Saccharomyces cerevisiae* (KM504287.1) amounted to 65.80 ± 0.52% of the theoretical ethanol yield. Overall, for the first time, the present work reports the production of extremozymatic (thermo, alkali-, and halo-stable) cellulase from *N. synnemataformans* NBRM9. Therefore, this strain is recommended for use as a biotool in many lignocellulosic-based applications operating under harsh conditions.

## 1. Introduction

Lignocellulosic agricultural wastes are the most prevalent renewable biomass on Earth. They are composed of the three main components of plant materials—cellulose, hemicellulose, and lignin [1]. Tons of these lignocellulosic wastes are annually generated in forestry, agriculture, and agro-industrial operations. As a result, lignocelluloses have been the focus of interest for researchers around the world who are trying to develop natural biomass-based technologies to reduce the dependence on exhaustible and expensive sources. Additionally, it serves as a readily available, economically viable, and renewable feedstock for a variety of lignocellulosic-based applications [2].

Cellulose is the main component of lignocellulosic waste and accounts for 40–55% of its dry weight. It is the most abundant biopolymer in nature and consists of β-D-glucose molecules linked by β-1,4-glycosidic bonds. The degradation of the lignocellulosic biomass can occur through a variety of chemical, physical, and enzymatic mechanisms. Enzyme-based hydrolysis is the best-known process because it has a high specificity, does not produce toxic substances, and does not cause substrate loss [3]. Lignocellulolytic enzymes are among the most powerful enzymes produced by microorganisms and can be extensively used in a variety of lignocellulose-based enterprises; these enzymes include cellulases, hemicellulases, and lignolytic enzymes [4].

Cellulases have been important biocatalysts for many decades. They have been shown to have potential biotechnological applications in a variety of industrial bioprocesses, including in the textile industry as biopolishing agents, in the pulp and paper industry as bio-modifying agents, in the laundry and detergent industry as bio-accelerators for washing processes, in agriculture as biocontrol agents, in food manufacturing as bio-clarification agents, in feed production to improve the nutrient digestibility of feed, in biofuel industries as saccharifying agents, and in medicine as anti-biofilm agents [5],[6].

More than 3,000 enzymes have been discovered, most of which are used in industrial and biotechnological applications; however, the enzyme market is still too small to meet the needs of the industrial sector. The main reason for the insufficient demand for enzymes is that many of the currently available enzymes are not adaptable to many industrial environments because they are not able to tolerate industrial operating conditions [7], such as pressure, heat, salinity, alkalinity, and acidity, while ensuring high conversion rates and reproducibility [8]. Additionally, enzymes are used in technologies that often employ harsh processing conditions [9]. Therefore, highly reproducible biocatalysts that can withstand fluctuating conditions such as pressure, temperature, pH, salinity, and other physicochemical parameters are needed for industrial operation. A crucial strategy to overcome the technical and financial drawbacks of ordinary enzymes, which has gained importance in recent years, is the switch from ordinary to extremozymes [10]. Extremozymes have been adapted to work under harsh physical-chemical conditions, and their use in various industrial applications has increased. Understanding the specific factors that confer the ability to withstand extreme habitats on such enzymes has become a priority for their biotechnological use [11].

Extremozymes are biocatalysts produced by extremophilic microorganisms that survive under extreme conditions. To date, enzymes from such microorganisms have had a major impact on biotechnology from commercial and economic perspectives [12],[13]. Therefore, the use of extremophilic microorganisms or their cellulases for the pretreatment of lignocellulosic material is attracting much attention in current research due to the numerous advantages they offer in the industrial field [14]. Lignocellulosic extremozymes have a wide range of applications, including in the textile, detergent, paper and pulp, biofuel, pharmaceutical, fine chemical, food, and feed industries. While the detergent, textile, paper and pulp industries require thermoalkaliphilic lignocellulosic enzymes, the biofuel, pharmaceutical, food, and feed industries are looking for thermostable and acidophilic enzymes to depolymerize lignocellulosic biomass [15]. In addition, enzymatic hydrolysis can be carried out directly with thermostable enzymes after heating without the need for a precooling step. This saves energy, shortens the process time, reduces the risk of contamination, increases substrate solubility and mass transfer rates, improves saccharification yield and, as a result of all these factors, makes the process more feasible and economical [16]. Therefore, lignocellulosic extremozymes are highly desirable for industrial applications, and the production of a variety and large quantities of such microbial enzymes is of great interest. Microbial cellulases are produced by various soil organisms, such as bacteria, actinomycetes, and fungi. Among soil microorganisms, actinomycetes are among the most important bacterial groups, known as suppliers, of important industrial enzymes with various biotechnological applications [17].

The phylum Actinobacteria is a distinct taxonomic group within the bacterial kingdom. Members of this phylum are gram-positive, highly GC-containing, often branched, and filamentous organisms that are widespread in nature, with most of them usually found in soil; they are particularly important because of their crucial role in recycling dead organic materials [18]. The cellulolytic activity from actinomycetes has been reported in many genera, including the following: *Streptomyces* [17]; *Streptosporangium*, *Dactylosporangium*, *Kitasatospora*, *Asanoa*, and *Nonomurae* [19]; *Thermobifida* and *Micromonospora* [20]; *Cellulomonas* and *Thermomonospora* [21]; and the genus *Nocardiopsis* [22]. Members of the genus *Nocardiopsis* belong to the phylum Actinomycetota, class Actinobacteria, order Actinomycetales, and family Nocardiopsaceae. Like most other actinomycetes, species of the genus *Nocardiopsis* are generally found in soil. Different soil types (desert, alkaline, and saline) have produced several novel species with unique thermophilic, alkaliphilic, and halophilic natures, and many species of this genus can produce various types of extremozymes [23].

Based on the crucial information available from genomic and protein sequencing data of actinomycetes, different common genera have been documented to produce a wide array of ordinary enzymes; however, the exploration of extremozymes with novel specificities, especially from uncommon genera, remains a priority of ongoing research. The aim of this study is to investigate the cellulolytic activity of soil-isolated actinomycetes, evaluate their ability to utilize various cellulosic agro-industrial wastes for cellulase production, optimize certain physicochemical factors for enzyme production, determine the activity and stability of partially purified cellulase (PPC), and evaluate its biotechnological applications in bioethanol production, as well as its antibiofilm activity.

## 2. Materials and methods

### 2.1. Culture media

The carboxy methyl cellulose (CMC) agar medium used for cellulase screening was purchased from Merck, Darmstadt, Germany. The Mueller-Hinton broth (MHB) medium used for the anti-biofilm assay was purchased from Oxoid (UK). Other culture media used in this study were prepared from analytical-grade ingredients purchased from Himedia laboratories (Mumbai, India) and Sigma–Aldrich (USA).

### 2.2. Cellulosic raw materials

Four lignocellulosic agro-industrial wastes, namely bean straw (BS), rice straw (RS), sawdust (SD), and wheat straw (WS), were collected from local fields in Egypt and used as cellulosic substrates. These raw materials were previously washed with distilled water, air-dried, ground, and sieved through a 0.2-mm sieve before being stored.

### 2.3. Isolation of actinomycetes

#### 2.3.1. Soil sampling and pretreatment

A total of thirty-six sandy desert soils (∼200 g each) were collected on the 4^th^–10^th^ of October 2022 from four different governorates, namely Rafha, Al-Markooz, Al-Owiqela, and Al-Kasb, in the northern border region of the Kingdom of Saudi Arabia (data of the collected soils are presented in Table S1). After transport to the laboratory, the soils were sieved to exclude large particles and then selectively pretreated by air-drying at room temperature for 7 days, heating to 70 °C for 20 min, and mixing with CaCO_3_ (1 g/100 g soil) for 24 h [24].

#### 2.3.2. Isolation and cultivation of actinomycete cultures

For the isolation and cultivation of actinomycetes, a soil suspension was prepared from each pretreated soil, and then serial dilutions (10^−1^–10^−5^) were performed. An aliquot of 0.1 mL of each dilution was inoculated onto International *Streptomyces* Project No. 4 (ISP–4, inorganic salt–starch agar) medium (g/L: soluble starch 10.0, (NH_4_)_2_SO_4_ 2.0, MgSO_4_.7H_2_O 1.0, K_2_HPO_4_ 1.0, NaCl 1.0, CaCO_3_.2H_2_O 2.0, agar 20, and distilled H_2_O up to 1 L, pH 7.2 ± 0.2). Then, the inoculated plates were incubated at 37 ± 2 °C for 7–14 days. Colonies with suspected actinomycete morphology were picked, purified by subculturing, and stored on ISP–4 slants at 4 °C.

### 2.4. Screening for cellulase-producing actinomycetes

#### 2.4.1. Preliminary screening on solid media

The isolated actinomycetes were inoculated in a circular pattern onto the surface of plates containing CMC media (g/L: CMC 10, yeast extract 5, peptone 5, K_2_HPO_4_.3H_2_O 1, MgSO_4_.7H_2_O 0.2, agar 20, and distilled H_2_O up to 1 L, pH 7.0 ± 0.2); then, the plates were incubated at 37 ± 2 °C for 5 days. The detection of cellulase activity was performed using Gram's iodine method, in which the plates were flooded with a 0.33% iodine solution for 5 min. The clear zone formed around the actinomycete colonies was used as an indicator of cellulase activity. The tests were performed in triplicate, and the cellulolytic index (CI) was calculated according to Ferbiyanto et al. [25] using Eq (1).

$$\text{Cellulolytic Index}\left(\text{CI}\right)=\frac{\text{Diameter of clear zone}-\text{Diameter of actinomycete colony }}{\text{Diameter of actinomycete colony}}$$ (1)

#### 2.4.2. Screening for cellulase production on liquid media

The cellulase-producing actinomycetes selected from the preliminary screening were tested for the production of the enzyme on liquid media using the modified method of Kshirsagar et al. [26]. In brief, fresh seed suspensions of the selected actinomycetes were prepared by inoculating three cork borer disks (6 mm diameter) taken from a 7-day-old culture (grown on ISP–4 plates) into a 250 mL Erlenmeyer flask containing 100 mL ISP–4 broth medium. The inoculated flasks were incubated in a rotary shaker at 150 rpm and 37 ± 2 °C for 72 h. Then, 100 µL of each suspension (adjusted to 1 × 10^5^ CFU/mL) was inoculated into a 500-mL flask containing 100 mL of modified Dubos salt (MDS) liquid medium (g/L: K_2_HPO_4_ 1.0, NaNO_3_ 0.5, KCl 0.5, MgSO_4_.7H_2_O 0.5, FeSO_4_.7H_2_O 0.001, and distilled H_2_O up to 1 L, pH 7.2 ± 0.2), supplemented with 20 g/L CMC as the sole carbon source. The flasks were incubated for 5 days at 37 ± 2 °C and shaken at 150 rpm. After incubation, the culture broth was filtered through a Whatman filter (0.45 µm) and centrifuged at 10,000 × g for 20 minutes under refrigeration. The resulting cell-free supernatants (CFS) containing the crude cellulase were harvested and stored at 4 °C.

#### 2.4.3. Quantification of cellulase activity

The quantification of crude cellulase produced in the harvested CFS of each isolate was performed by estimating the reducing sugar content using the 3,5-dinitrosalicylic (DNS) acid method [27]. In a test tube, 0.5 mL of CFS was mixed with 0.5 mL of 1% (w/v) CMC (dissolved in 0.05 M sodium citrate buffer, pH 4.8), and the mixture was incubated in a water bath at 50 °C for 30 min. At the end of the incubation, the reaction was stopped by adding 1.5 mL of 3,5-DNS and incubated at 100 °C for 10 min. After cooling, the activity of the reaction mixture was measured with a spectrophotometer at 575 nm and compared with that of the control. Using the calibration curve for glucose (Figure S1), a unit of enzyme activity was determined as the amount of enzyme that released 1 µM glucose/mL/min. The enzyme activity (U/mL/min) was calculated using the following Eq (2):

$$\text{Enzyme activity}(\text{U}/\text{ml})/\text{min}=\frac{(\text{Concentration of glucose}(\text{mg}/\text{ml})\times \text{reaction volume })\;}{\left(\text{Molecular weight of glucose}\times \text{reaction time}\times \text{volume of enzyme}\times \text{dilution factor}\right)}$$ (2)

#### 2.4.4. Screening for cellulase production using agro-industrial wastes

The agro-industrial wastes BS, RS, SD, and WS were oven-dried at 60 °C for 48 h, crushed to a fine powder, passed through a 600 µm mesh filter, and used as a carbon source in MDS broth media (pH 7.2 ± 0.2) for cellulase production under submerged fermentation (SmF) conditions. A fresh seed suspension (prepared as mentioned above, adjusted to 1 × 10^5^ CFU/mL) of the highest cellulase-producing actinomycete NBRM9 was inoculated into a 250 mL Erlenmeyer flask that contained 100 mL of MDS liquid medium supplemented with 2% (w/v) agro-waste and incubated for 7 days at 37 ± 2 °C with shaking at 150 rpm. After fermentation, the culture broth was filtered, and the resulting CFS was stored at 4 °C and quantitatively analyzed for activity.

### 2.5. Identification of the most potent actinomycete NBRM9

#### 2.5.1. Cultural and morphological characteristics

To study the culture characteristics, the NBRM9 isolate was allowed to grow on seven International *Streptomyces* Project (ISP) and other growth media at 37 ± 2 °C for 7, 14, and 21 days. The culture features, including the degree of growth, the color of the aerial and substrate mycelia, and the presence of diffusible pigments, were visually examined and recorded following the guidelines adopted by Shirling and Gottlieb [28]. The micromorphological characteristics were investigated by examining spore-bearing hyphae and entire spore chains under a light microscope (400 ×) using the coverslip culture technique, in which the isolate was inoculated onto the bottom of a cover slip dipped in an ISP–2 (yeast extract-malt extract agar) plate and incubated at 37 ± 2°C for 10–14 days. To examine the spore surface morphology, cells of the NBRM9 isolate (cultured on ISP–2 plates at 37 ± 2°C for 10 days) were fixed by mixing with glutaraldehyde (2.5%) for 1 h, refixed in osmium tetroxide (2%) for 30 min, and dehydrated with a graded dilution of ethanol (25%–85%) for 5 min each. The dehydrated sample was coated with gold-palladium and examined at different magnifications using a scanning electron microscope (SEM, JEOL JSM 5400, Japan) [29]. The morphology of the spore surface was examined at different magnifications using a scanning electron microscope (SEM). Then, the recorded cultural and micromorphological characteristics were compared with those in Bergey's Manual of Systematic Bacteriology [30],[31].

#### 2.5.2. Chemotaxonomic analyses

After cultivation of the NBRM9 isolate on ISP–4 broth medium at 37 ± 2 °C in a rotary shaker at 150 rpm for 72 h, the biomass (50 mg) was collected by centrifugation at 6000 × g for 10 min, dried well overnight at 45 °C, and analyzed for chemotaxonomic characteristics according to the method of Lechevalier and Lechevalier [32]. Detection of the type of a diaminopimelic acid (DAP) isomer (LL– or *meso*–DAP) was performed by paper chromatography of 10 mg of dried biomass hydrolyzed in 6 N HCl. The pattern of whole-cell sugar was determined by thin-layer chromatography of the dried biomass (5 mg) hydrolyzed in 1 N H_2_SO_4_.

#### 2.5.3. Physiological and biochemical characteristics

The physiological and biochemical characteristics, including the ability of the NBRM9 isolate to utilize different carbon and nitrogen sources, grow at different temperatures (25–60 °C), pH values (5–13), NaCl concentrations (1–10%), inhibitors (0.1% phenol, 0.01% sodium azide, 0.001% crystal violet, and different antibiotics), and degrade keratin, esculin, pectin, starch, Tween 80, and xanthine were determined. Additionally, the amylolytic, chitinolytic, lipolytic, pectinolytic, and proteolytic activities were tested using tryptic soy agar (TSA) media supplemented with starch (0.65%, w/v), chitin (1%, w/v), glycerol tributyrate (1%, v/v), pectin (1%, w/v), and milk powder (1%, w/v), respectively. All experiments were recorded after 7 days of incubation at 37 ± 2°C and studied according to the established methods described by Williams et al. [33].

#### 2.5.4. 16S rRNA gene sequencing and phylogenetic analyses

For molecular identification of the NBRM9 isolate, its DNA was extracted using the modified method of Miller et al. [34]. Briefly, separate colonies of the isolate were taken from five-day-old cultures plated on ISP–4 medium and suspended in 100 µL of sterile deionized water. The prepared suspension was incubated at 95 °C in a water bath for 15 min and centrifuged at 15,000 × g for 10 min. Thereafter, the DNA-containing cell lysate was separated. The 16S rRNA gene was amplified by PCR using a genomic DNA template and two universal bacterial primers: 27f (5-AGAGTTGATCCTGGCTCAG-3) and 1492r (5-GGTTACCTTGTTACGACTT-3) [35]. Fifty microliters of the PCR mixture was prepared by mixing 1 µL of extracted genomic DNA, 0.5 mM MgCl_2_, 1 x PCR buffer, 0.25 mM deoxynucleotide triphosphate (dNTPs), 2.5 U of Taq polymerase (QIAGEN), and 0.5 µM of each primer. The PCR was performed in a thermal cycler with a 3-min hot start at 94 °C, 30 cycles at 94 °C for 30 s, 55 °C for 30 s, and 72 °C for 1 min, followed by gene extension for 10 min at 72 °C. Automated sequencing was performed using an ABI 3730x1 DNA sequencer at the GATC Company (Germany) according to previously published procedures [36].

Alignment of the obtained 16S rRNA gene sequence with previously published bacterial 16S rRNA sequences in the NCBI database was performed using the GenBank search tool on the Centre's BLAST website (http://www.ncbi.nlm.nih.gov/BLAST). The phylogenetic tree was inferred using the neighbor-joining method with a bootstrap test (1,000 replicates) in the MEGA11 software.

### 2.6. Statistical optimization of cellulase production by the NBRM9 isolate

The optimal conditions for cellulase production were investigated using a response surface methodology (RSM) based on a four-variable/three-level Box–Behnken design (BBD) that included temperature, pH, incubation time, and substrate concentration. One hundred microliters of an NBRM9 spore suspension (1 × 10^5^ CFU/mL) was used to inoculate 250 mL Erlenmeyer flasks with 75 mL of MDS broth medium containing different concentrations of bean straw (1, 3 and 5%, w/v) and incubated under shaking conditions (150 rpm) at different tested temperatures (30, 40 and 50) for different incubation times (3, 5 and 7 days) and different pH values (5, 7 and 9). Twenty-seven experiments with central points were used to fit the polynomial pattern based on a Box–Behnken design (BBD, 4 variables) created using the Minitab 18® software. A three-level and four-factor experimental BBD was investigated, and the number of tests (N) was determined according to Eq (3):

N=(2k×(k−1)+C0) (3)

where *k* is the number of factors and *C0* is the number of central points, which is equal to 3. The impact of variables on the simulation (Y) was determined by employing a second-order polynomial equation that was utilized to predict the idealistic states of cellulase biosynthesis:

Y=β0+βAA+βBB+βCC+βDD+βAAA2+βBBB2+βCCC2+βDDD2+βABAB+βACAC+βADAD+βBCBC+βBDBD+βCDCD

where *Y* is the response variable, *β0* is the intercept, *βA, βB, βC*, and *βD* are linear coefficients, *βAA, βBB, βCC*, and *βDD* are square coefficients, *βAB, βAC, βAD, βBC, βBD*, and *βCD* are interaction coefficients, and *A, B, C, D, A2, B2, C2, D2, AB, AC, AD, BC, BD*, and *CD* are the levels of independent variables. Minitab 18® was utilized to determine the coefficients of the variables, the interaction variables, and the contour plots. An analysis of the regression equation and the creation of response graphs were used to determine the ideal values of the tested variables.

### 2.7. Partial purification of the produced cellulase

After incubation of the NBRM9 isolate under the determined optimal production conditions (run no. 26), the fermentation broth was collected (2.5 L total volume) and centrifuged at 10,000 × g for 20 min under refrigeration. The obtained CFS that contained crude cellulase was partially purified by precipitation with cold acetone at different ratios (1:1–1:6 v/v). After an overnight incubation at –20°C, the precipitates were collected by centrifugation at 10,000 × g for 15 min and resuspended in a 0.05 M sodium citrate buffer (pH 4.8). The cellulase activity and protein concentration were both measured in the CFS and in the collected precipitates [37]. Eq (4–6) were applied to calculate the specific activity (U/mg), yield (%), and purification fold of the partially purified cellulase:

$$\text{Specific activity}(\text{U}/\text{mg})=\frac{\text{Total activity}}{\text{Total protein }}$$ (4)

$$\text{Yield}\left(\text{\%}\right)=\frac{\text{Total units in partially purified enzyme }}{\text{Total units in crude enzyme }}\times 100$$ (5)

$$\text{Purification fold}=\frac{\text{Specific activity of partially purified enzyme }}{\text{Specific activity of crude enzyme}}$$ (6)

### 2.8. Characterization of partially purified cellulase (PPC)

#### 2.8.1. Effect of different parameters on the activity and stability of PPC

The optimal temperature for the activity of the PPC was determined in the range of 20–90 °C, and the thermal stability was determined after the enzyme was preincubated at each temperature for 1 h before screening. The ideal pH for the enzyme activity was determined at the optimal temperature using different pH buffers with values between 3 and 11. The pH stability was evaluated after the enzyme had been stored at these pH values for one hour prior to screening. Additionally, the activity of the PPC was evaluated at different concentrations of NaCl (1–19%), and its stability was determined after the enzyme was preincubated at each concentration for 1 h before being evaluated at the optimal temperature and pH. Furthermore, the activity of the enzyme was investigated in the presence of various metal ions (Ca^2+^, Fe^2+^, K^+^, Mn^2+^, Mg^2+^, Na^2+^, NH^3+^, and Zn^2+^, 10 mM each) and after treatment with various detergents at different concentrations, including hydrogen peroxide, Tween 20 and urea (1% and 5%, v/v), Na_2_CO_3_ (50 and 75 mM), sodium dodecyl sulfate (SDS), sodium lauryl sulfate (SLS), and ethylenediaminetetraacetic acid (EDTA) sodium (5 and 10 mM, w/v). Moreover, the stability of the enzyme was evaluated when the enzyme was kept with these ions and detergents for 1 h under optimal conditions of temperature, pH, and salinity. The activity values obtained were compared with those of the control enzyme (100% activity).

#### 2.8.2. Cellulase‑substrate specificity and kinetics

The effect of substrate concentration on the activity of the PPC was determined by measuring the activity at different concentrations (0.25–2.5%) of CMC under the optimal assay conditions. The kinetic parameters of the Lineweaver–Burk plot were used to calculate the Michaelis–Menten constant (kmKm) and maximum enzyme velocity (V_max_) using Eq (7) of Lineweaver et al. [38]:

1/V=Km/Vmaxx1/[S]+1/ Vmax (7)

where Km is the Michaelisian constant (g/L), S is the substrate concentration (g/100 mL), V is the starting rate (g/100/mL/min), and the maximum velocity (V_max_) values of the enzymes were estimated from the slope and intercept of the straight Lineweaver–Burk plot.

#### 2.8.3. Cellulase immobilization and SEM

PPC immobilization was performed following the method of Al Mousa et al. [39]. Briefly, the enzyme solution was mixed with an equal volume of a 3.0% sodium alginate solution. Calcium alginate beads were formed by adding the mixture dropwise to CaCl_2_ (0.2 M) at 4 °C. The calcium alginate beads were rinsed with double distilled H_2_O to remove any unscattered enzyme units. Then, the beads were dried and stored in phosphate buffer. The entrapped beads were activated with glutaraldehyde to covalently bind cellulase to the beads. The external surface shape of the calcium alginate beads before and after cellulase immobilization was examined by SEM. Free and immobilized cellulase were estimated, and the yield of immobilization was determined according to Eq (8):

$$\text{Immobilization yield}\left(\text{Y\%}\right)=\frac{\text{Activity of immobilized enzyme }}{\text{Activity of soluble enzyme}}\times 100$$ (8)

### 2.9. Evaluation of the biotechnological applications of PPC

#### 2.9.1. In vitro anti-biofilm activity

The anti-biofilm activity of the PPC was evaluated against four multidrug-resistant (MDR), biofilm-forming gram-positive (*Enterococcus faecium* TS7 and *Staphylococcus aureus* WS12) and gram-negative (*Acinetobacter baumannii* SC6 and *Pseudomonas aeruginosa* UC22) test strains [40] using 96-well flat-bottomed polystyrene microtiter plates according to the modified method of Kamali et al. [5]. Briefly, individual wells of a sterile microtiter plate were filled with 90 µL of MHB medium and inoculated with 10 µL of an overnight bacterial suspension (1 × 10^5^ CFU/mL). To the mixture, 10 µL of cellulase at concentrations of 0.62, 1.25, 2.5, 5, and 10 U/mL was added individually to each well. A negative control (well with MHB without bacteria or enzyme) and a positive control (well with MHB and bacteria without enzyme treatment) in additional wells with MHB-inoculated bacteria treated with heat-inactivated (HI, 100 °C) cellulase as a second positive control were all examined. After 24 hours of incubation at 37 °C, the biofilms that formed in each well were fixed in alcohol, stained with 0.1% (w/v) crystal violet for 30 min, and then air-dried. Then, the optical density (OD) was determined using a microplate reader at 595 nm. The percentage of inhibition of biofilm formation was calculated using Eq (9):

$$\text{Biofilm inhibition}\left(\text{\%}\right)=1-\frac{\text{OD}595\text{ of cells treated with different concentration of cellulase}}{\text{OD}595\text{ of non treated control}}\text{X}100$$ (9)

#### 2.9.2. Bioethanol production using BS hydrolysate

After 8 days of incubation with the NBRM9 isolate, the BS hydrolysate resulting from cellulolytic hydrolysis was subjected to fermentation and bioethanol production by the sugar-fermenting *Saccharomyces cerevisiae* strain KM504287.1. One mL of a freshly prepared yeast seed suspension (1 × 10^5^ CFU/mL) grown on yeast extract-peptone dextrose medium was used to ferment 100 mL of BS hydrolysate in 250 ml Erlenmeyer flasks. After incubation of the inoculated flasks for 72 h (12 intervals) in a rotary shaker at 150 rpm and 30 ± 2 °C, the fermented samples were centrifuged at 10,000 rpm for 10 min. The colorimetric DNS method was used to determine the residual total reducing sugar (TRS) content, and redox titration was used to determine the concentration of bioethanol produced [41]. The theoretical ethanol yield was calculated using the modified Eq (10) of Gunasekaran and Kamini [42]:

$$\text{Theoretical ethanol yield}\left(\text{\%}\right)=\frac{\text{Produced ethanol}\times 100}{\text{TRS}}$$ (10)

### 2.10. Data analysis

All experiments were performed in triplicate, and the data are presented as the mean ± standard deviation (SD). Significant differences were determined using the SPSS software (version no. 18) with one-way analysis of variance (ANOVA), and the difference was considered to be statistically significant if p ≤ 0.05.

## 3. Results

### 3.1. Selection of cellulase-producing actinomycetes

During our screening for cellulase-producing actinomycetes, a total of thirty-one (NBR1–NBR31) actinomycete cultures with different morphologies were isolated from soil samples collected from different locations in the desert of the northern border region of Saudi Arabia. The isolated actinomycetes were screened for their cellulolytic activity on solid CMC plates. Among them, twelve isolates showed different cellulolytic indices (ranging from 0.55 ± 1.15 to 3.26 ± 0.66) and were selected as cellulase-producing actinomycetes (Table S2), with the highest CI value (3.26 ± 0.66) for the NBRM9 isolate. Testing the selected isolates to utilize CMC substrate as the sole carbon source in the liquid medium showed that only five isolates (41.66%) had cellulase activity (Figure S2), with the highest activity (2.59 U/mL) detected for the NBRM9 isolate.

### 3.2. Cellulase production using agro-industrial wastes

**Figure 1.**
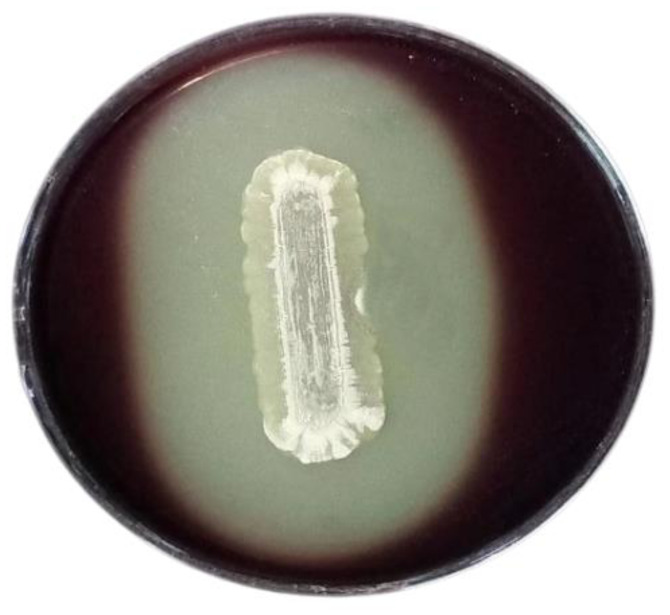
CMC agar plate with cellulase activity showing a clear zone formed by the most effective isolate NBRM9.

The most effective isolate, NBRM9 (Figure 1), which showed the highest cellulolytic activity in both the solid and liquid screenings, was selected and tested for its ability to produce cellulase from various agro-industrial wastes under SmF. The results showed that this isolate exhibited various degrees of cellulolytic activity, with 2.57 ± 0.24, 2.76 ± 0.07, 3.64 ± 0.13, and 5.50 ± 0.20 U/mL in the presence of RS, SD, WS, and BS, respectively (Figure S3); bean straw displayed the highest cellulase productivity (5.50 ± 0.20 U/mL).

### 3.3. Identification of the actinomycete isolate NBRM9

#### 3.3.1. Conventional identification

**Figure 2.**
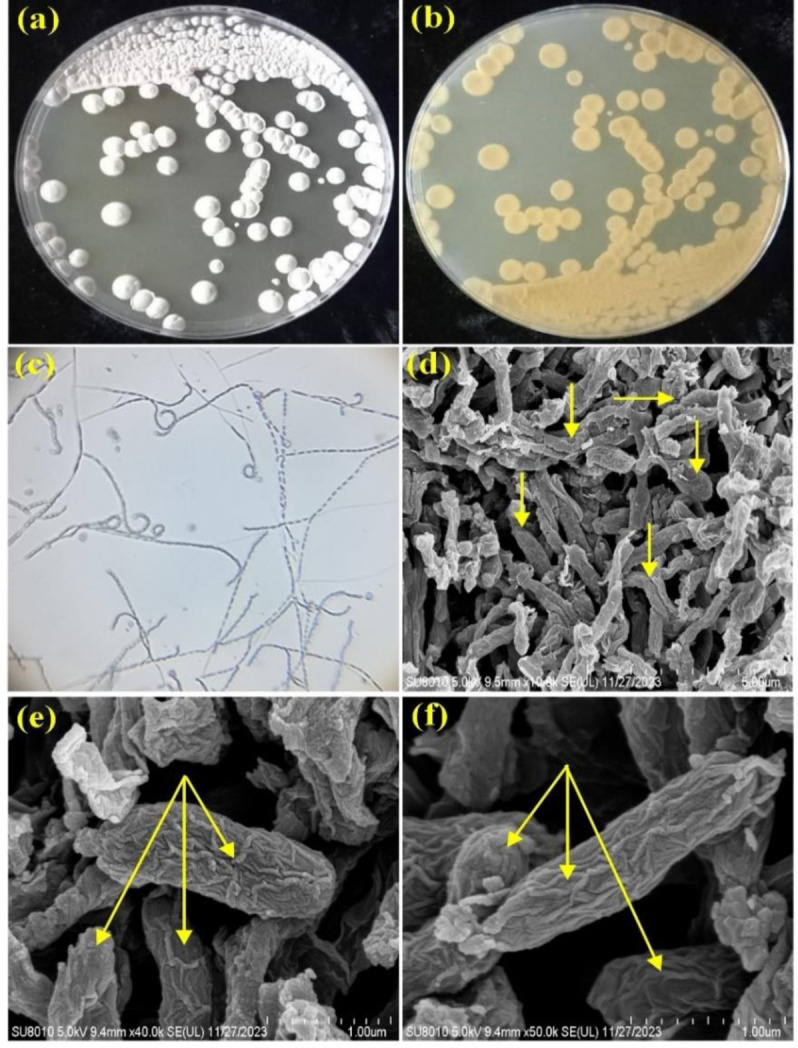
Cultural and morphological characteristics of the actinomycete isolate NBRM9. (a) Color of aerial mycelium, (b) color of substrate mycelium (both colors were recorded for growth of the isolate on ISP–2 medium), (c) hyphae of aerial mycelium bearing moderately branched, spiral and zig-zag-shaped spore chains seen under light microscopy (400×), (d) SEM micrograph (10,000×) showing threads of spore chains wrapping together, and the synnemata formed (marked with light arrows) at magnifications of 40,000× (e) and 50,000× (f).

The recorded culture characteristics of the actinomycete NBRM9 (Table S3) showed that the organism abundantly grew on all media tested, except ISP–3, where it did not grow. The color of the mature aerial mycelium was white–yellow (Figure 2a), while the color of the substrate mycelium was pale yellow (Figure 2b). Diffusible pigments, including melanin, were not detected. The morphological characteristics of this NBRM9 isolate grown on ISP–2 medium were examined by light microscopy (400 ×), which revealed moderately branched, spiral, and zigzag-shaped aerial mycelium (Figure 2c), and by SEM (10,000×), which showed threads wrapping together, some of which were divided into elongated spores with smooth surfaces (Figure 2d), while others formed synnemata that could be clearly seen at magnifications of 40,000× (Figure 2e) and 50,000 × (Figure 2f).

Chemotaxonomic analyses of whole-cell hydrolysates of the NBRM9 isolate revealed that this isolate contained *meso*-2,6-diaminopimelic acid (*meso*-DAP), and no diagnostic sugars were detected, thus indicating that the cell wall peptidoglycan belongs to type III (wall-chemo type III); therefore, this isolate has the same chemical composition as that of the genus *Nocardiopsis*. The results of the physiological and biochemical characteristics of the NBRM9 isolate (Table 1) showed that the isolate exhibited strong growth on various carbon sources, especially CMC, galactose, and lactose. Additionally, KNO_3_ and urea were utilized as the best nitrogen sources for good growth. The extremophilic growth properties were noted for the isolate because of its ability to grow in a wide range of environmental conditions, including pH (5–13, with an optimum at 9–11), temperature (25–60 °C, with an optimum at 35–50 °C), and tolerance to NaCl (up to 10%, with an optimum up to 7%), thus indicating the extremophilic nature of this isolate. Promising enzymatic activities were observed for this isolate, especially for amylase, cellulase, and pectinase. This information could be useful for future work to tune the medium to achieve higher yields of the desired metabolites. A summary of these results and other tests can be found in Table 1.

**Table 1.** Physiological and biochemical characteristics of the NBRM9 isolate.

Characters	Growth degree	Characters	Growth degree
Melanin pigment production		Tolerance to NaCl (%)
Liquid tryptone yeast extract	−	1–7	+++
Tyrosine agar	−	8	++
Peptone yeast extract iron agar	−	9	+
Utilization of carbon sources		10	Wg
CMC	++	Degradation ability
Fructose	+	Citrate	+
Galactose	+++	Esculin	+
Glucose	+	Pectin	++
Lactose	+++	Tyrosine	+
L-rhamnose	+	Urea	++
Mannose	+	Xanthine	++
Pectin	++	Enzymatic activities
Starch	++	Amylase	+++
Utilization of nitrogen sources		Catalase	++
KNO_3_	+++	Cellulase	+++
Malt extract	++	Chitinase	+
NaNO_3_	++	Gelatinase	+
(NH_4_)_2_SO_4_	+	Pectinase	+++
Peptone	++	Protease	++
Urea	+++	Tolerance to growth inhibitors (%)
Yeast extract	+	Crystal violet (0.0001)	+
Growth at different pH values		Potassium cyanide (0.001)	+
5–8	+	Sodium azide (0.02)	++
9–11	+++	Phenol (0.1)	+
12	++	Sensitivity to antibiotics
13	Wg	Gentamicin (30)	R
Growth at different temperature (°C)		Rifampicin (50)	R
25–30	++	Vancomycin (50)	S
35–50	+++	Streptomycin (50)	R
55	+	Penicillin G (10 IU)	S
60	Wg	Clarithromycin	S

*Symbols of growth degree: − (negative), + (moderate), ++ (good), +++ (abundant), Wg (weak growth). R (resistant), S (sensitive).

#### 3.3.2. Molecular identification

**Figure 3.**
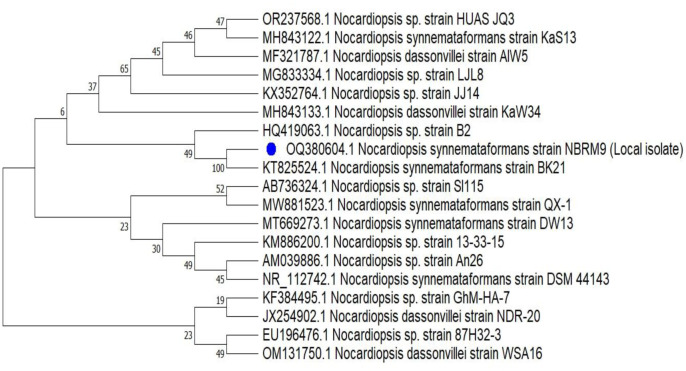
Phylogenetic tree of the *Nocardiopsis synnemataformans* strain NBRM9 inferred using the neighbor-joining method in MEGA 11.0 software. This tree shows the relationships between the NBRM9 isolate and closely linked species of the genus *Nocardiopsis*. The percentage of replicate trees in which the associated taxa clustered together in the bootstrap test (1000 replicates) is shown next to the branches. The evolutionary distances were computed using the p-distance method and are in units of the number of base differences per site. This analysis involved 19 nucleotide sequences. There were a total of 1175 positions in the final dataset.

The conventional identification of the actinomycete NBRM9 was confirmed by the molecular phylogeny of the sequence of the 16S rRNA gene (1393 bp), which was deposited in the GenBank database under the accession number OQ380604.1. The neighbor-joining phylogenetic tree (Figure 3) revealed that the isolate belongs to a single, unique subclade with *Nocardiopsis synnemataformans* strain BK21 (GenBank accession number KT825524.1), with which it shares 99.93% 16S rRNA gene sequence similarity. NBRM9 is quite identical to *Nocardiopsis synnemataformans*; hence, it was given the name *N. synnemataformans* NBRM9.

### 3.4. Statistical optimization of cellulase production using RSM

To achieve maximum cellulase production by *N. synnemataformans* NBRM9, the application of RSM based on a four-variable/three-level BBD for optimizing analytical methods is presented in this study. The independent factors and their competency levels used in the optimization of cellulase production are listed in Table S4. The BBD of the independent factors along with the predicted and experimental values are shown in Table S5. The predicted production of cellulase was calculated as follows:

Cellulase (U/mL) = 24.30 - 0.244 A - 1.279 B - 2.609 C + 3.810 D - 0.00043 A2 + 0.0703 B2 + 0.1100 C2- 0.345 D2 + 0.01708 AB - 0.00048 AC + 0.0180 AD + 0.1636 BC - 0.5414 BD + 0.2289 CD

**Figure 4.**
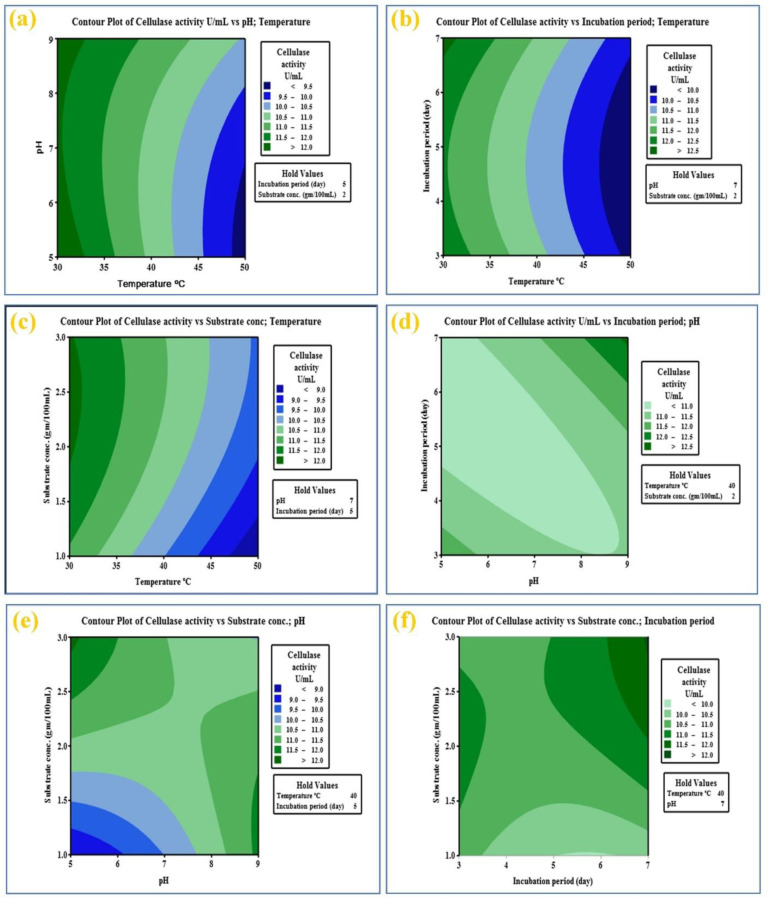
Contour plot showing interactions between independent variables: (a) incubation temperature with pH, (b) incubation temperature with incubation period, (c) incubation temperature with substrate concentration, (d) pH with incubation period, (e) pH with substrate concentration, (f) incubation period with substrate concentration for cellulase productivity by *N. synnemataformans* NBRM9.

The highest cellulase activity of *N. synnemataformans* NBRM9 (13.20 U/mL) was obtained in run no. 26 at an incubation temperature of 40 °C, pH of 9, incubation time of 7 days, and BS concentration of 2 g/100 mL. The results of the ANOVA for the quadratic cellulase model are shown in Table S6. The model terms with a *p* value < 0.05 were considered significant. The model's F value for cellulase production (31.56) showed that the model was significant. A value of “Prob > F” < 0.05 indicated that the model terms were significant. For cellulase activity, C, D, B2, C2, D2, AC, AD, BC, and CD were significant model terms. An F value of 227.29 for lack of fit indicated that the lack of fit was not significant in terms of pure error. A nonsignificant lack of fit is appropriate to consider the model fit. The multiple correlation coefficient R^2^ = 0.9779 for cellulase indicated a favorable association between the experimental and predicted values and illustrated the model accuracy with an improved response. The regression values agreed with the fitted and predicted R^2^ values. Contour plots explaining the relationships between parameters and defining the optimal scale of each factor for cellulase activities are represented in Figure 4.

### 3.5. Cellulase purification

The cellulase produced by *N. synnemataformans* NBRM9 was partially purified from its CFS using various concentrations of acetone, and the highest cellulase activity was obtained by precipitating CFS with acetone at a 1:4 ratio. Cellulase purification resulted in a 3.39-fold purification, an 18.96% cellulase recovery, and a specific activity of 69.48 U/mg (Table 2).

**Table 2.** Summary of the specific activity, yield and purification fold of cellulase produced by *N. synnemataformans* NBRM9.

Purification steps	Volume (mL)	Total activity (U/L)	Total protein (mg/L)	Specific activity (U/mg)	Yield (%)	Purification (fold)
CFS	1000	9303.91 ± 29.40	45356.74 ± 59.50	20.52 ± 0.05	100 ± 0.0	1.0 ± 0.0
Acetone	100	1764.18 ± 52.57	25391.70 ± 80.68	69.48 ± 2.21	18.96 ± 0.56	3.39 ± 0.10

The data are presented as the average of three replicates (mean ± SD).

### 3.6. Characterization of PPC

#### 3.6.1. Activity and stability of PPC

**Figure 5.**
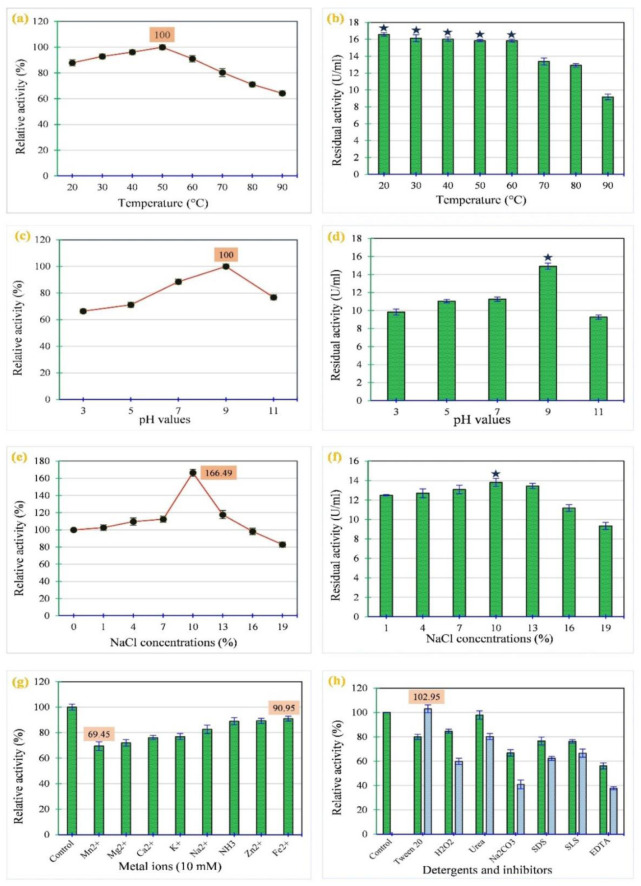
Impact of different parameters on the activity and stability of PPC produced by *N. synnemataformans* NBRM9. (a) Activity at different temperatures (20–90°C), (b) Thermal stability, (c) Activity at different pH values (3–11), (d) pH stability, (e) Activity at different NaCl concentrations (0–19%), (f) Salinity stability, (g) Activity in the presence of different metal ions, and (h) Activity in the presence of different concentrations of detergents and inhibitors (green columns are the high concentrations, blue the low ones). In this figure, the values represent the mean ± SD of triplicate experiments (n = 3, p < 0.05), and 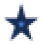 is a marker for the stability of PPC.

Various parameters, including temperature, pH, NaCl%, metal ions, detergents, and inhibitors, were investigated to characterize the activity and stability of the PPC in the presence of these conditions (Figure 5). Regarding the effect of temperature on the activity and stability of the PPC (Figure 5a, b), it was found that the enzyme was active in the temperature range of 20–90 °C and exhibited optimal (relative) activity (of 100%, with 18.13 ± 0.08 U/mL) at 50 °C. Additionally, it showed a broad thermal stability in the range of 20 to 60°C, with a residual activity ranging from 100% to 95.79 ± 1.06%. Regarding its activity and stability in the pH range (Figure 5c, d), it showed activity at different pH values (3–11), showing an optimal (relative) activity (of 100%, with 16.75 ± 0.13 U/mL) at pH 9.0. However, its stability was determined at pH 9.0 with a residual activity of 100%; the stability gradually decreased below and above this value. With respect to the impact of salinity on the activity and stability of the PPC (Figure 5e, f), cellulase was found to be active over a wide NaCl range (1–19%). Its activity and stability were NaCl concentration dependent, where they increased in the range of 1 to 13%, with the optimal (relative) activity (166.49 ± 0.17%, with 14.10 ± 0.45 U/mL) and stability (99.95 ± 0.78%) achieved at 10% NaCl.

After incubation of the PPC with the different metal ions, a significant (p ≤ 0.05) inhibitory effect on cellulase activity was observed for all metals tested (Figure 5g), with the highest inhibitory effect observed for Mn^2+^ (relative activity of 69.45 ± 0.56%) and the smallest effect for Fe^2+^ with a relative activity of 90.95 ± 0.11%. Furthermore, the activity of the PPC significantly (p ≤ 0.05) decreased in the presence of low and high concentrations of the detergents and inhibitors tested (Figure 5h), showing a relative activity between 37.71 ± 0.61% and 97.93 ± 0.45%, except for the high concentration (5%) of Tween 20, which increased the activity to 102.95 ± 0.35% compared to the activity of the control (100%). There was also a significant difference (p ≤ 0.0) at low and high concentrations of these metal ions, detergents, and inhibitors.

#### 3.6.2. Cellulase kinetics

**Figure 6.**
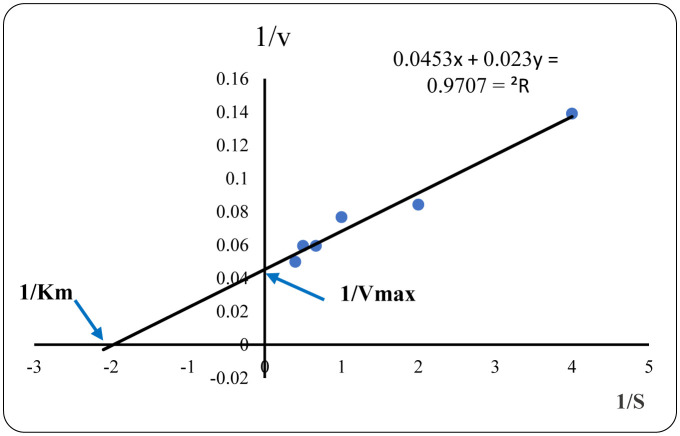
The relationship between the CMC concentration (S) and cellulase velocity (V) is represented through the Lineweaver–Burk plot by plotting 1/substrate concentration (1/S) vs. 1/enzyme velocity (1/V). The values represent the mean ± SD of triplicate experiments (n = 3, p < 0.05).

The cellulase substrate specificity was investigated, and the results showed that the cellulase activity gradually increased as the substrate concentration increased from 0.25% to 2.5%, where the maximum cellulase activity was reached at a CMC concentration of 2.5%. Based on the Lineweaver–Burk plot (Figure 6), which shows the relationship between the enzyme velocity (V_max_) and the substrate concentration, the maximum value of V_max_ was 20.19 ± 1.88 U/mL. The value of Km is inversely proportional to the affinity of the enzyme for the substrate. The Km was therefore calculated to be 0.25 ± 0.07 mM. The lower Km value obtained shows that the PPC produced by *N. synnemataformans* NBRM9 has a strong affinity for its CMC substrate.

#### 3.6.3. Cellulase immobilization

**Figure 7.**
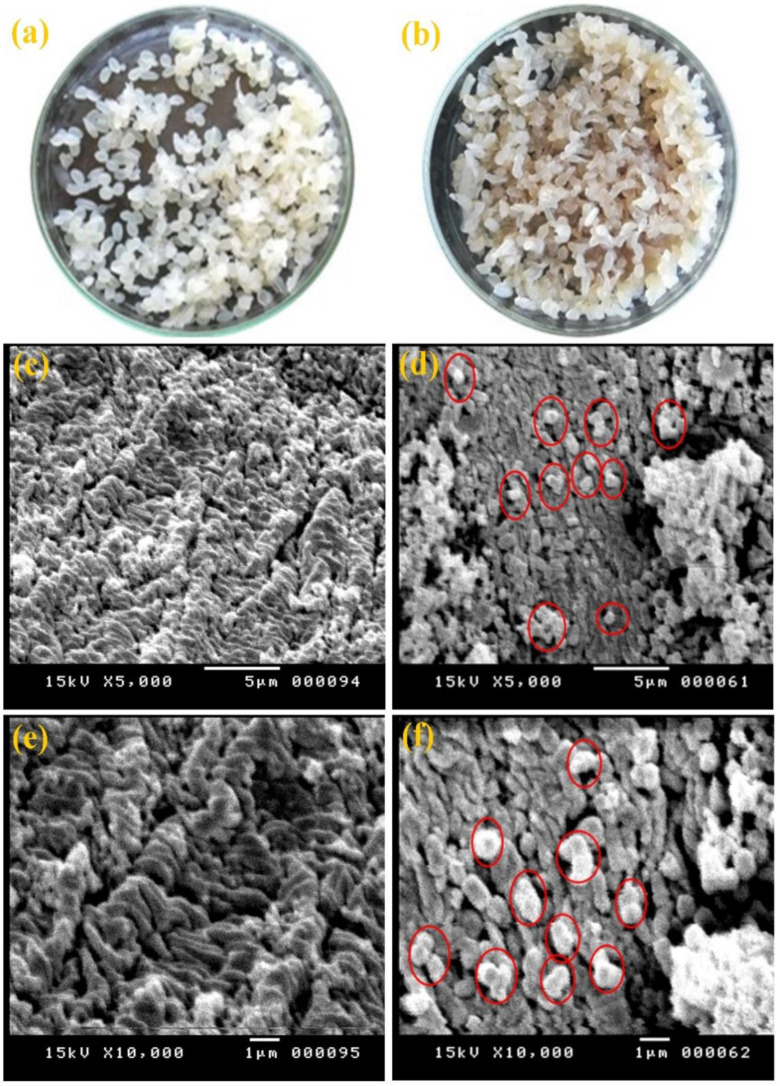
Immobilization of PPC produced by *N. synnemataformans* NBRM9. (a–b) Visual examination of calcium alginate beads, where (a) enzyme-free beads (control) and (b) beads were treated with PPC. (c–d) SEM images of calcium alginate beads at low magnification (5,000×), where (c) enzyme-free beads (control) and (d) beads with immobilized PPC. (e–f) SEM images of calcium alginate beads at high magnification (10,000×), where (e) enzyme-free beads (control) and (f) beads with immobilized PPC. In this figure, the red circles refer to the cellulase entrapped on the surface of the calcium alginate beads.

The PPC produced by *N. synnemataformans* NBRM9 was immobilized using calcium alginate beads, thus resulting in an immobilization yield of 69.58 ± 0.13%, which demonstrated its high reusability. In the SEM images (Figure 7) of the calcium alginate beads without entrapped cellulase, the openings of the calcium alginate beads were visible, while the openings were coated with intense molecular cellulase.

### 3.7. Biotechnological applications of PPC

#### 3.7.1. In vitro anti-biofilm activity

**Figure 8.**
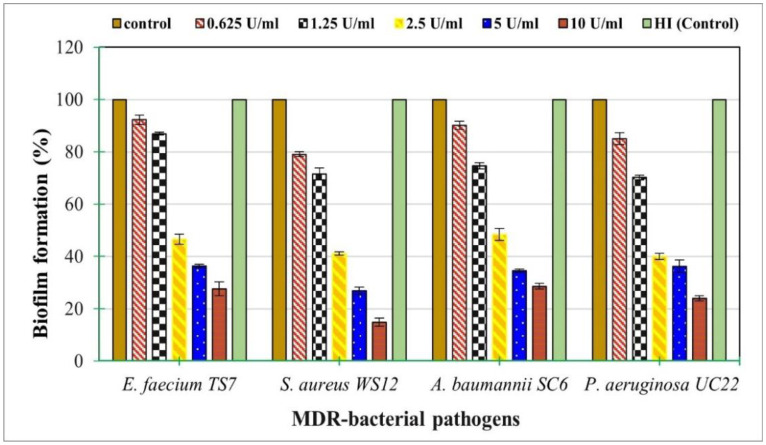
Dose-dependent inhibitory effect of PPC from *N. synnemataformans* NBRM9 on the biofilm formation of MDR bacterial pathogens growing on MHB media. The control was enzyme-free medium, while HI (control) was medium supplemented with heat-inactivated (HI) cellulase. The values represent the mean ± SD of triplicate experiments (n = 3, p < 0.05).

The results of the in vitro anti-biofilm ability of the PPC produced by *N. synnemataformans* NBRM9 against four MDR bacterial pathogens (Figure 8) demonstrated that the PPC significantly (p < 0.05) reduced biofilm formation in a concentration-dependent manner for the four species tested. The highest biofilm inhibition was observed for *S. aureus* WS12, with a reduction between 20.93 ± 0.93% and 85.15 ± 1.60% at concentrations of 0.625 and 10 U/mL, respectively. In contrast, the lowest reduction was observed for *A. baumannii* SC6, with an inhibition ranging from 9.94 ± 1.56% to 71.45 ± 1.10% at concentrations of 0.625 and 10 U/mL, respectively. A moderate anti-biofilm ability was observed against *P. aeruginosa* UC22, followed by *E. faecium* TS7.

#### 3.7.2. Bioethanol fermentation

**Figure 9.**
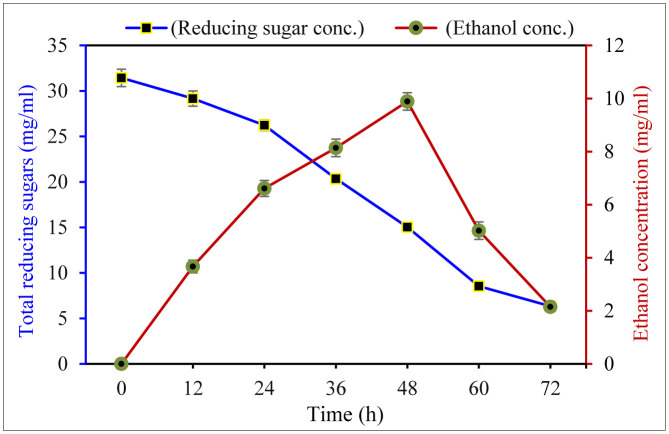
Reducing sugar content and ethanol yield of bean straw hydrolysate after fermentation by *S. cerevisiae* KM504287.1.

The results of the fermentation of BS hydrolysate by *S. cerevisiae* KM504287.1 at different incubation times (Figure 9) showed that BS hydrolysate had a moderate sugar content, with the TRS determined before fermentation (at time zero) being 31.43 ± 0.12 mg/mL. After fermentation, the TRS content significantly decreased and reached its lowest value (2.34 ± 0.02 mg/mL) after 72 h. At the same time, ethanol production also significantly increased over time, reaching maximum concentrations of 9.89 ± 0.05 mg/mL and 65.80% of the theoretical ethanol yield after 48 h of incubation.

## 4. Discussion

For a long time, enzymes have been of vital importance to our daily lives due to their valuable properties and diverse applications. They are essential ingredients in the production of numerous industrial products, such as food and beverages, animal feed, pharmaceuticals, therapies, diagnostics, personal care products, leather, detergents and textiles, pulp and paper, chemicals, and biofuels, among others [43]. According to a report by BBC Research, the global market for enzymes in industrial applications is expected to increase from $6.4 billion in 2021 to $8.7 billion in 2026, at a compound annual growth rate (CAGR) of 6.3% from 2021 to 2026 [44].

Among industrial enzymes, cellulases have been ranked as the third most important enzymes in industrial markets [45]. In view of the increasing demand for cellulases and the resulting requirement for higher productivity, the search for cellulases with new specificities and applications is crucial. In this respect, the selection of microorganisms and their production efficiency are of an utmost importance for the continuation of successful industrial operations. Researchers around the world are intensively searching for novel bioactive metabolites, including enzymes from a variety of microbial sources, including actinomycetes [17],[19],[46],[47]. In addition, there is growing interest in isolating actinomycetes from extreme environments (e.g., deserts and Antarctica) to study the adaptability of novel strains and the metabolites produced by these microorganisms for various industrial purposes [48],[49].

In our study, five cellulase-producing isolates were selected from thirty-one soil-derived actinomycetes, which were tested for their cellulolytic activity via solid and liquid assays. Although the determined number of cellulase-producing actinomycetes was low, it was relatively similar to that reported by Daquioag and Penuliar [17], but lower than that reported by Djebaili et al. [50], which we attribute to the different locations and environments of the actinomycetes studied.

According to Dasgupta and Brahmaprakash [51], the physicochemical properties of the soil and the structure and functions of the microbial community are influenced by location and environment. The present study focused on the northern border region, which is located in the northern Kingdom of Saudi Arabia. This region covers an area of approximately 127,000 km² and includes many hills, valleys, and plains. The desert of this region is an extreme environment, as it is characterized by special conditions, such as dry, hot summers (50–55 °C on average), cold winters (below 0 °C), little rainfall, high alkalinity, and some salinity.

Wilson and Brimble [52] reported that microbes must first adapt and evolve to withstand various stresses. As a result, they have the potential to evolve new bioactive metabolites that exert specific functions and activities. Therefore, our actinomycete isolates from the northern border region soil are extremophilic organisms and represent an unexplored source for the discovery of useful bioactive metabolites. To our knowledge, we are the first group to investigate the cellulolytic activity of extremophilic actinomycetes in this area.

Of the screenings performed, the actinomycete NBRM9 was the most potent; therefore, it was tested for cellulase production using four inexpensive and readily available lignocellulosic wastes. Of these, BS was the best and recorded the highest cellulase productivity at 5.50 ± 0.20 U/mL. BS is the vegetative part of faba beans (*Vicia faba* L.) and is widely cultivated worldwide. Approximately 90% of faba bean cultivation occurs in the European Union, Asia, and Africa, including Egypt [53]. Therefore, it serves as a readily available, economically viable, and renewable lignocellulosic feedstock for a variety of agro-industrial-based applications.

One of the comparable results of this study is that the selected NBRM9 isolate showed low cellulase activity (2.59 U/mL) when CMC was used as a substrate, while its activity significantly increased (5.50 ± 0.20 U/mL) when BS was used. We attribute this to the water hydrophobicity, high crystallinity, complexity, and high polymerization of cellulose, which make it difficult for bacteria to be attacked by enzymes. In contrast, natural cellulosic wastes such as BS exhibit low polymerization and have a variety of functional groups on their surface, which facilitates the attachment of bacterial cells and increases their utilization; similar results were reported by Yousef and Mawad [54].

Polyphasic identification of the NBRM9 isolate involved various conventional and molecular approaches. The results of the conventional identification showed that this isolate had a white–yellow mass color, zigzag aerial mycelium, and wall-chemo type III, all of which are consistent with some members of the genus *Nocardiopsis*, based on Lechevalier and Lechevalier [32] and Bergey's Manual of Systematic Bacteriology [30],[55]. Moreover, the results of physiological and biochemical characteristics showed that this isolate has optimal growth conditions (pH 9–11, temperature 35–50 °C and 1–7% salinity) in the extreme growth range, which classifies it as an alkaliphilic, thermophilic, and halophilic strain, which is a promising outcome and is consistent with that of some of these *Nocardiopsis* species.

Bennur et al. [23] reported that members of the genus *Nocardiopsis* generally inhabit extreme environments, including desert sands and saline, hypersaline, and alkaline habitats. Combining the results of conventional and molecular identification revealed that the isolate NBRM9 is identical to the species *Nocardiopsis synnemataformans* [30]. Similarly, Saratale and Oh [22] reported the cellulolytic activity of *Nocardiopsis* sp. KNU, which was isolated from soil and identified based on morphological, physiological, and biochemical characteristics, in addition to 16S rRNA sequencing. There are few reports on the cellulolytic activity of *Nocardiopsis* spp. isolated from various sources; however, the cellulase activity of *Nocardiopsis synnemataformans* has not yet been reported. Therefore, the present study is the first report on the extremophilic, soil-isolated *N. synnemataformans* NBRM9 as a cellulase-producing actinomycete. Another promising finding of the current study is that this strain has a unique enzymatic activity for several commercially important enzymes, including amylase, chitinase, pectinase, and protease, in addition to cellulase, which increases the industrial applicability of this strain. Therefore, the discovery of such an actinomycete is the most important result of our study. Actinomycetes are a great source of enzymes with novel specificities and applications [48],[56].

Due to the increasing demand for cellulase in a variety of industrial processes, reducing production costs by optimizing growth and production conditions is one of the most important goals of cellulase research. Using BS as a substrate, cellulase production by *N. synnemataformans* NBRM9 was statistically optimized, reaching a maximum activity (13.20 U/mL) at 40 °C, pH 9, 7 days of incubation and a substrate concentration of 2%. Compared with the nonoptimized medium, the optimized medium resulted in an approximately threefold increase in cellulase production. Based on previous studies dealing with the production of cellulase using lignocellulosic agro-wastes from different microbial sources, we acknowledge that the determined productivity is relatively low even after optimization, which we attribute to the lignocellulosic BS used in the present study without any prior treatment methods.

There are very few reports on the production of cellulolytic enzymes using low-cost agro-industrial wastes by *Nocardiopsis* species; however, they have not yet been reported from soil-derived *N. synnemataformans*. In general, the optimal parameters determined are relatively similar to those in a previous study by Saratale and Oh [22], in which the maximum production of thermotolerant and alkali-tolerant cellulase by soil-isolated *Nocardiopsis* sp. KNU was measured at 37 °C and a pH of 6.5 for 8 days using 1 g/100 mL rice straw as the substrate. Furthermore, Walker et al. [57] reported that indoor isolated and alkali-tolerant *Nocardiopsis* sp. SES28 has an optimal cellulase activity at a pH of 8.0. The other parameters studied have not yet been reported in combination.

To verify the applicability of the PPC as a potential candidate for bio-industrial processes, especially in harsh operation, its activity and stability were investigated in the presence of various physicochemical parameters. Yousef and Mawad [54] reported that the operating conditions of enzymes, including temperature, pH, salinity, and additives, played a crucial role in their activity and stability. Another promising finding of the current study is the unique properties of the PPC produced by *N. synnemataformans* NBRM9, including its wide range of activity (20–90 °C, 3–11 pH, 1–19% NaCl), as well as its stability (with >95% residual activity) at 50 °C, pH 9.0, and 10% NaCl, thus demonstrating its extremozyme nature. To our knowledge, these combined activities have not yet been reported for bacterial cellulases, especially for cellulases produced by actinomycetes.

Earlier studies reported on the activity and stability of bacterial cellulase under different conditions. The thermos/alkali-tolerant cellulase from *Nocardiopsis* sp. KNU has an optimal activity at 40 °C and pH 5.0, and it retained >55% of its activity at pH 10 and 40 °C [22]. In another study by Walker et al. [57], alkali-tolerant cellulase (β-1,4-glucanase) produced by *Nocardiopsis* sp. SES28 showed activity in the pH range of 6.5–10.0, with an optimal value at 9.0; it retained 94% of its activity at pH 10. Moreover, the thermostable/halostable cellulase from *Virgibacillus salarius* BM-02 was found to be active and stable at 40–60 °C, 6–12% NaCl, and a pH of 5–8 [54]. Many *Nocardiopsis* spp. are alkaliphilic and thermophilic actinomycetes that can produce various types of alkalophilic and/or alkali-tolerant or thermostable enzymes, such as cellulase, xylanase [22], protease [58], chitinase [59], and amylase [60]. For the first time, the present work reports the production of extremozymatic (thermo, alkali-, and halo-stable) cellulase from *Nocardiopsis synnemataformans*.

Regarding the kinetics of cellulase, the low Km value (0.25 ± 0.07 mM) shows that cellulase has a strong affinity for its CMC substrate. This is the amount of substrate needed to reach half of the maximum initial velocity [61]. Many bacterial cellulases with different Km values have been reported. These included cellulase from *Bacillus subtilis* TD6 [62], with a Km value of 2.9 mg/mL, cellulase from *Virgibacillus salarius* BM-02, with a Km value of 2.1 mM [54], and cellulase from *Streptomyces albuduncus*, with a Km value of 92.30 mg/mL [63]. Thus, the enzyme-substrate affinity varies with different enzyme sources and with the substrate used for estimation.

For the hydrolytic degradation of cellulases, the immobilization of cellulases has proven to be a successful method to maintain their reusability [64], with such immobilized cellulases retaining their activity over a longer period of time and being able to be reused up to four times. In the present study, the PPC produced from *N. synnemataformans* NBRM9 was immobilized using calcium alginate beads, thus resulting in an immobilization yield of 69.58 ± 0.13%. Bangrak et al. [65] reported that calcium alginate beads are among the most commonly used carriers for enzyme immobilization. Many different types of immobilized cellulases have been reported, including cellulase from *Pseudomonas stutzeri* KDPM2, which has 80% relative activity [66], cellulase from *Mucor circinelloides*, which has 84.02 ± 0.63% relative activity [39], and cellulase from *Bacillus* sp., which has 90% relative activity [67].

Concentration-dependent anti-biofilm activity was recorded for the PPC against some MDR bacterial pathogens. It is important to note that the bacterial test organisms used in this experiment were MDR species belonging to the ESKAPE (*Enterococcus faecium*, *Staphylococcus aureus*, *Klebsiella pneumoniae*, *Acinetobacter baumannii*, *Pseudomonas aeruginosa* and *Enterobacter* sp.) pathogens. ESKAPE are known as the key pathogens involved in biofilms and chronic wound infections [68]. Infections caused by these ESKAPE pathogens are the leading causes of morbidity and mortality around the world [69]; therefore, the search for natural anti-biofilm agents against such pathogens is of a great importance. Actinomycetes are a rich source of bioactive metabolites with diverse pharmacological and medicinal activities, including anti-biofilm effects [40],[70]. The use of microbial-derived cellulases as an alternative approach to combat biofilms has already been reported for various microbial sources, such as bacterial cellulase from *Bacillus subtilis* strain Fatma/1, which showed 84.61% anti-biofilm activity against *Pseudomonas aeruginosa* [71], and fungal cellulase (combined with amylase and protease) from *Penicillium janthinellum* EU2D-21, which showed 88.76%, 87.42%, 85.5%, and 79.72% anti-biofilm activities against the bacterial species *Pseudomonas aeruginosa*, *Staphylococcus aureus*, *Escherichia coli*, and *Salmonella enterica*, respectively [72].

Compared to the abovementioned literature, our anti-biofilm results revealed that at the highest concentration tested (10 U/mL), the PPC had promising anti-biofilm activity ranging from 72.43 ± 2.62 to 85.15 ± 1.60%, while at a lower concentration (2.5 U/mL), it was also effective at inhibiting more than 50% of biofilms formed by MDR-ESKAPE pathogens. To our knowledge, there are very few reports on the anti-biofilm activity of bacterial cellulase against some bacterial pathogens but not yet against MDR-ESKAPE pathogens. Therefore, the PPC produced by *N. synnemataformans* NBRM9 is a potential alternative antibiofilm approach, especially for MDR pathogens.

A high residual (TRS) sugar content was recorded for BS hydrolysate resulting from the hydrolytic action of the PPC produced by *N. synnemataformans* NBRM9. The use of the yeast *S. cerevisiae* strain KM504287.1 in bioethanol fermentation showed a promising fermentative efficiency in the conversion of TRS in BS hydrolysate to ethanol, thus yielding 65.80 ± 0.52% after 48 h of fermentation. Our results suggest that cellulase-hydrolyzed bean (faba bean) straw is a good lignocellulosic biomass for ethanol production by *S. cerevisiae*. Similar results were reported by Petersson et al. [73], who reported that *S. cerevisiae* produced 52% ethanol during the simultaneous enzymatic hydrolysis of field bean straw. In another study by Saratale and Oh [22], rice straw was hydrolyzed by *Nocardiopsis* sp. KNU, thus resulting in a theoretical ethanol yield of 86% after 48 h of fermentation by *S. cerevisiae*. Although the ethanol yield obtained was greater than that in the current study, we attribute this to two factors: first, the agricultural lignocellulosic wastes used in that study were subjected to physical and chemical treatments that were not performed in our study; and second, there were also differences in the production potential of the yeast strains used. From these results, it can be concluded that fermentation with a subsequent enzymatic hydrolysis by *N. synnemataformans* NBRM9 is an effective method for the production of fermentable sugars that can be used for bioethanol production without any physical or chemical treatment of agricultural waste. The present study has a few limitations. First, cellulase-inducing substrates should be expanded to more than four substrates to evaluate the actual cellulase production potential of strain NBRM9. Second, when studying the optimization of production conditions, other parameters need to be investigated, such as the size of the inoculum of the producer strain, which could increase the productivity. Finally, the shelf life of the PPC should be investigated to verify the applicability of this enzyme in biotechnological processes.

## 5. Conclusions

For the first time, the extremophilic *Nocardiopsis synnemataformans* strain NBRM9 was reported as a soil-dwelling actinomycete. This strain could utilize many lignocellulosic agro-wastes as cellulase-inducing substrates without any prior treatments. Therefore, this actinomycete is recommended for use as a biotool in many lignocellulosic-based applications. The PPC produced from this strain exhibited novel and promising thermo, alkali- and halo-stable specificities, thus classifying it as an extremozyme and encouraging its use in industrial bioprocesses operating under harsh conditions. In addition to its unique properties, it exhibited promising biotechnological applicability as an anti-biofilm agent, especially against multidrug-resistant bacterial pathogens, and as a biocatalyst in the hydrolysis of natural cellulosic feedstocks for bioethanol fermentation. In view of the aforementioned promising results, future work will conduct a more in-depth analysis of the genome of this strain to reveal the molecular mechanism of its production of extreme enzymes and/or investigate its possible application in other bioindustrial processes. Comprehensively, this study emphasizes that the soils on the northern border of Saudi Arabia are a valuable source of extremophilic actinomycetes with a variety of bioactive metabolites of industrial importance.

## Use of AI tools declaration

The authors declare they have not used Artificial Intelligence (AI) tools in the creation of this article.



## References

[1] Limayem A, Ricke SC (2012). Lignocellulosic biomass for bioethanol production: current perspectives, potential issues and future prospects. Prog Energy Combust Sci.

[2] Saini A, Aggarwal K, Sharma A (2015). Actinomycetes: a source of lignocellulolytic enzymes. Enzyme Res.

[3] Nwamba MC, Song G, Sun F (2021). Efficiency enhancement of a new cellulase cocktail at low enzyme loading for high solid digestion of alkali catalyzed atmospheric glycerol organosolvent pre-treated sugarcane bagasse. Bioresour Technol.

[4] Sun Y, Cheng J (2002). Hydrolysis of lignocellulosic materials for ethanol production: a review. Bioresour Technol.

[5] Kamali E, Jamali A, Izanloo A (2021). In vitro activities of cellulase and ceftazidime, alone and in combination against *Pseudomonas aeruginosa* biofilms. BMC Microbiol.

[6] Jayasekara S, Ratnayake R, Pascual A.R., Martín M.E.E. (2019). Microbial cellulases: an overview and applications. Cellulose.

[7] Irwin JA, Baird AW (2004). Extremophiles and their application to veterinary medicine. Ir Vet J.

[8] Haki GD, Rakshit SK (2003). Developments in industrially important thermostable enzymes: A review. Bioresour Technol.

[9] Díaz-Tena E, Rodríguez-Ezquerro A, López De Lacalle Marcaide LN (2013). Use of extremophiles microorganisms for metal removal. Procedia Eng.

[10] Amobonye A, Bhagwat P, Singh S (2021). Plastic biodegradation: frontline microbes and their enzymes. Sci Total Environ.

[11] Dalmaso GZL, Ferreira D, Vermelho AB (2015). Marine extremophiles: a source of hydrolases for biotechnological applications. Mar Drugs.

[12] Jin M, Gai Y, Guo X (2019). Properties and applications of extremozymes from deep-sea extremophilic microorganisms: a mini review. Mar Drugs.

[13] Raddadi N, Cherif A, Daffonchio D (2015). Biotechnological applications of extremophiles, extremozymes and extremolytes. Appl Microbiol Biotechnol.

[14] Dumorné K, Córdova DC, Astorga-Eló M (2017). Extremozymes: a potential source for industrial applications. J Microbiol Biotechnol.

[15] Gündüz Ergün B, Çalık P (2016). Lignocellulose degrading extremozymes produced by *Pichia pastoris*: current status and future prospects. Bioprocess Biosyst Eng.

[16] Turner P, Mamo G, Karlsson EN (2007). Potential and utilization of thermophiles and thermostable enzymes in biorefining. Microb Cell Fact.

[17] Daquioag JEL, Penuliar GM (2021). Isolation of actinomycetes with cellulolytic and antimicrobial activities from soils collected from an urban green space in the Philippines. Int J Microbiol.

[18] Al-Shaibani MM, Mohamed RMSR, Sidik NM (2021). Biodiversity of secondary metabolites compounds isolated from phylum Actinobacteria and its therapeutic applications. Molecules.

[19] Putri AL, Setiawan R (2019). Isolation and screening of actinomycetes producing cellulase and xylanase from Mamasa soil, West Sulawesi. IOP conference series: earth and environmental science.

[20] Zhang F, Chen JJ, Ren WZ (2011). Cloning, expression and characterization of an alkaline thermostable GH9 endoglucanase from *Thermobifida halotolerans* YIM 90462 T. Bioresour Technol.

[21] Kuhad RC, Gupta R, Singh A (2011). Microbial cellulases and their industrial applications. Enzyme Res.

[22] Saratale GD, Oh SE (2011). Production of thermotolerant and alkalotolerant cellulolytic enzymes by isolated *Nocardiopsis* sp. KNU. Biodegradation.

[23] Bennur T, Kumar AR, Zinjarde S (2015). *Nocardiopsis* species: incidence, ecological roles and adaptations. Microbiol Res.

[24] Seong CN, Choi JH, Baik KS (2001). An improved selective isolation of rare actinomycetes from forest soil. J Microbiol.

[25] Ferbiyanto A, Rusmana I, Raffiudin R (2015). Characterization and identification of cellulolytic bacteria from gut of worker macrotermes gilvus. HAYATI J Biosci.

[26] Kshirsagar SD, Bhalkar BN, Waghmare PR (2017). Sorghum husk biomass as a potential substrate for production of cellulolytic and xylanolytic enzymes by *Nocardiopsis* sp. KNU. 3 Biotech.

[27] Miller GL (1959). Use of dinitrosalicylic acid reagent for determination of reducing sugar. Anal Chem.

[28] Shirling EB, Gottlieb D (1966). Methods for characterization of *Streptomyces* species. Int J Syst Bacteriol.

[29] Tresner HD, Davies MC, Backus EJ (1961). Electron microscopy of *Streptomyces* spore morphology and its role in species differentiation. J Bacteriol.

[30] Goodfellow M, Kämpfer P, Busse H-J (2012). Bergey's Manual of Systematic Bacteriology, Volume 5: The Actinobacteria, part A.

[31] Williams S, Sharpe M, Holt J (1989). Bergey's Manual of Systematic Bacteriology Vol. 4.

[32] Lechevalier MP, Lechevalier H (1970). Chemical composition as a criterion in the classification of aerobic actinomycetes. Int J Syst Bacteriol.

[33] Williams ST, Goodfellow M, Alderson G (1983). Numerical classification of *Streptomyces* and related genera. J Gen Microbiol.

[34] Miller DN, Bryant JE, Madsen EL (1999). Evaluation and optimization of DNA extraction and purification procedures for soil and sediment samples. Appl Environ Microbiol.

[35] Lane D, Stackebrandt E., Goodfellow M. (1991). 16S/23S rRNA sequencing. Nucleic Acid Techniques in Bacterial Systematic.

[36] Sanger F, Nicklen S, Coulson AR (1977). DNA sequencing with chain-terminating inhibitors. Proc Natl Acad Sci USA.

[37] Wood E (1991). Protein purification methods: a practical approach. Biochem Educ.

[38] Lineweaver H, Burk D, Deming WE (1934). The dissociation constant of nitrogen-nitrogenase in *Azotobacter*. J Am Chem Soc.

[39] Al Mousa AA, Abo-Dahab NF, Hassane AMA (2022). Harnessing *Mucor* spp. for xylanase production: statistical optimization in submerged fermentation using agro-industrial wastes. Biomed Res Int.

[40] El-Sayed MH, Alshammari FA, Sharaf MH (2023). Antagonistic potentiality of actinomycete-derived extract with anti-biofilm, antioxidant, and cytotoxic capabilities as a natural combating strategy for multidrug-resistant ESKAPE pathogens. J Microbiol Biotechnol.

[41] Pourkarim F, Rahimpour E, Khoubnasabjafari M (2020). A simple colorimetric method for determination of ethanol in exhaled breath condensate. Pharm Sci.

[42] Gunasekaran P, Kamini NR (1991). High ethanol productivity from lactose by immobilized cells of *Kluyveromyces fragilis* and *Zymomonas mobilis*. World J Microbiol Biotechnol.

[43] Ruiz-Villafán B, Rodríguez-Sanoja R, Sánchez S, Brahmachari G. (2023). Useful microbial enzymes—an introduction. Biotechnology of Microbial Enzymes: Production, Biocatalysis, and Industrial Applications, Second Edition.

[44] BCC Research. Global Enzymes Market in Industrial Applications, Report ID BIO030L, 2021.

[45] Pandey A, Selvakumar P, Soccol C (1999). Solid state fermentation for the production of industrial enzymes. Curr Sci.

[46] El-Sayed MH, Elsehemy IA (2017). *Paenibacillus* sp. strain NBR–10, a thermophilic soil-isolated bacterium with thermo-alkali stable pectinase activity. J Appl Environmnet Biol Sci.

[47] El-Sayed MH, Kobisi AA, Elsehemy IA (2023). Rhizospheric-derived *Nocardiopsis alba* BH35 as an effective biocontrol agent actinobacterium with antifungal and plant growth-promoting effects: in vitro studies. J Microbiol Biotechnol.

[48] Nazari MT, Machado BS, Marchezi G (2022). Use of soil actinomycetes for pharmaceutical, food, agricultural, and environmental purposes. 3 Biotech.

[49] Mohamed H, Hassane A, Rawway M (2021). Antibacterial and cytotoxic potency of thermophilic *Streptomyces werraensis* MI-S.24-3 isolated from an Egyptian extreme environment. Arch Microbiol.

[50] Djebaili R, Pellegrini M, Smati M (2020). Actinomycete strains isolated from saline soils: plant-growth-promoting traits and inoculation effects on *Solanum lycopersicum*. Sustainability.

[51] Dasgupta D, Brahmaprakash GP (2021). Soil microbes are shaped by soil physico-chemical properties: a brief review of existing literature. Int J Plant Soil Sci.

[52] Wilson ZE, Brimble MA (2009). Molecules derived from the extremes of life. Nat Prod Rep.

[53] Serafin-Andrzejewska M, Jama-Rodzeńska A, Helios W (2023). Accumulation of minerals in faba bean seeds and straw in relation to sowing density. Agric.

[54] Yousef NMH, Mawad AMM (2022). Characterization of thermo/halo stable cellulase produced from halophilic *Virgibacillus salarius* BM-02 using non-pretreated biomass. World J Microbiol Biotechnol.

[55] Williams S, Goodfellow M, Alderson G, Williams S., Sharpe M., Holt J. (1989). Genus *Nocardiopsis* Meyer 1976. Bergey's Manual of Systematic Bacteriology Vol. 4, 1^st^ Edition.

[56] Benhadj M, Gacemi-Kirane D, Menasria T (2019). Screening of rare actinomycetes isolated from natural wetland ecosystem (Fetzara Lake, northeastern Algeria) for hydrolytic enzymes and antimicrobial activities. J King Saud Univ - Sci.

[57] Walker D, Ledesma P, Delgado OD (2006). High endo-β-1,4-D-glucanase activity in a broad pH range from the alkali-tolerant *Nocardiopsis* sp. SES28. World J Microbiol Biotechnol.

[58] Moreira KA, Porto TS, Teixeira MFS (2003). New alkaline protease from *Nocardiopsis* sp.: partial purification and characterization. Process Biochem.

[59] Tsujibo H, Kubota T, Yamamoto M (2003). Characterization of chitinase genes from an alkaliphilic actinomycete, *Nocardiopsis prasina* OPC-131. Appl Environ Microbiol.

[60] Stamford TLM, Stamford NP, Coelho LCBB (2001). Production and characterization of a thermostable alpha-amylase from *Nocardiopsis* sp. endophyte of yam bean. Bioresour Technol.

[61] Tong CC, Cole AL, Shepherd MG (1980). Purification and properties of the cellulases from the thermophilic fungus *Thermoascus aurantiacus*. Biochem J.

[62] Andriani D, Sunwoo C, Ryu HW (2012). Immobilization of cellulase from newly isolated strain *Bacillus subtilis* TD6 using calcium alginate as a support material. Bioprocess Biosyst Eng.

[63] Harchand RK, Singh S (1997). Characterization of cellulase complex of *Streptomyces albaduncus*. J Basic Microbiol.

[64] Rajnish KN, Samuel MS, John J A (2021). Immobilization of cellulase enzymes on nano and micro-materials for breakdown of cellulose for biofuel production-a narrative review. Int J Biol Macromol.

[65] Bangrak P, Limtong S, Phisalaphong M (2011). Continuous ethanol production using immobilized yeast cells entrapped in loofa-reinforced alginate carriers. Braz J Microbiol.

[66] Desai MP, Pawar KD (2020). Immobilization of cellulase on iron tolerant *Pseudomonas stutzeri* biosynthesized photocatalytically active magnetic nanoparticles for increased thermal stability. Mater Sci Eng C Mater Biol Appl.

[67] Amadi OC, Awodiran IP, Moneke AN (2022). Concurrent production of cellulase, xylanase, pectinase and immobilization by combined cross-linked enzyme aggregate strategy- advancing tri-enzyme biocatalysis. Bioresour Technol Reports.

[68] Kadam S, Shai S, Shahane A (2019). Recent advances in non-conventional antimicrobial approaches for chronic wound biofilms: have we found the ‘chink in the armor’?. Biomedicines.

[69] Santajit S, Indrawattana N (2016). Mechanisms of antimicrobial resistance in ESKAPE pathogens. Biomed Res Int.

[70] Setiawati S, Yusan RT (2022). Actinomycetes as a source of potential antimicrobial and antibiofilm agents. Med Heal J.

[71] Ibrahim AM, Hamouda RA, El-Naggar NEA (2021). Bioprocess development for enhanced endoglucanase production by newly isolated bacteria, purification, characterization and in vitroin-vitro efficacy as anti-biofilm of *Pseudomonas aeruginosa*. Sci Rep.

[72] Nagraj AK, Gokhale D, Nagraj AK (2018). Bacterial biofilm degradation using extracellular enzymes produced by *Penicillium janthinellum* EU2D-21 under submerged fermentation. Adv Microbiol.

[73] Petersson A, Thomsen MH, Hauggaard-Nielsen H (2007). Potential bioethanol and biogas production using lignocellulosic biomass from winter rye, oilseed rape and faba bean. Biomass and Bioenergy.

