# Supplementary material for: Nocardiopsis synnemataformans NBRM9, an extremophilic actinomycete producing extremozyme cellulase, using lignocellulosic agro-wastes and its biotechnological applications
Source: AIMS Microbiol. 2024 Mar 12;10(1):187–219. doi: 10.3934/microbiol.2024010 (PMC10955166; doi:10.3934/microbiol.2024010)
Supplement: Supplementary file 1 [file microbiol-10-01-010-s001.pdf]

Microbiology

AIMS Microbiology, 10(1): 187–219.
DOI: 10.3934/microbiol.2024010
Received: 20 November 2023
Revised: 05 March 2024
Accepted: 06 March 2024
Published: 12 March 2024

http://www.aimspress.com/journal/microbiology

*Research article*

# *Nocardiopsis synnemataformans* NBRM9, an extremophilic actinomycete producing extremozyme cellulase, using lignocellulosic agro-wastes and its biotechnological applications

**Mohamed H. El-Sayed[1,2]*, Doaa A. Elsayed[1] and Abd El-Rahman F. Gomaa[3,4]**

[1] Department of Biology, College of Science and Arts, Northern Border University, Arar, Saudi Arabia
[2] Department of Botany and Microbiology, Faculty of Science, Al-Azhar University, Cairo 11884, Egypt
[3] Department of Botany and Microbiology, Faculty of Science, Al-Azhar University, Assiut Branch, Assiut 71524, Egypt
[4] The Key Laboratory of Biotechnology for Medicinal Plants of Jiangsu Province, School of Life Science, Jiangsu Normal University, Xuzhou, Jiangsu, 221116, PR China

* **Correspondence:** Email: Mohammed.Ahmed@nbu.edu.sa; Tel: +966545905650; Fax: +0000000000.

## Supplementary

**Table S1.** Geographical locations of sampling sites and properties of the collected soils.

| Location | Site No. | GPS | | Sample code | Number of samples | Sample characteristics |
|---|---|---|---|---|---|---|
| | | Latitude (N) | Longitude (E) | | | |
| Rafha | 1 | 43°31'30'N | 35°36'29'E | R-S1 | 4 | Yellow, sandy, dry, soft, and rough soil |
| | | 43°29'10'N | 34°39'29'E | R-S2 | 4 | |
| | | 43°32'52'N | 08°40'29'E | R-S3 | 4 | |
| Al-Markooz | 2 | 42°38'02'N | 35°07'30'E | M-S1 | 3 | Yellowish black, sandy, dry, soft soil |
| | | 42°38'10'N | 18°08'30'E | M-S2 | 2 | |
| | | 42°37'33'N | 14°08'30'E | M-S3 | 2 | |
| Al-Owiqela | 3 | 42°15'10'N | 56°19'30'E | O-S1 | 3 | Yellow, sandy, dry, rough soil |
| | | 42°15'26'N | 25°22'30'E | O-S2 | 2 | |
| | | 42°12'33'N | 44°22'30'E | O-S3 | 3 | |
| Al-Kasb | 4 | 41°55'37'N | 33°30'30'E | K-S1 | 4 | Yellow, sandy, dry, rough soil |
| | | 41°55'42'N | 05°30'30'E | K-S2 | 3 | |
| | | 41°56'31'N | 56°29'30'E | K-S3 | 2 | |

**Table S2**. Cultures of isolated actinomycetes and their cellulase activity on solid CMC media.

| Location/ Site No | Isolate code | Colony morphology* | | | | Cellulase activity* |
|---|---|---|---|---|---|---|
| | | Color | Form | Consistency | Pigment | |
| 1–S1 | NBRR1 | Medium gray | Irregular | Rough | ND | 0.0 |
| 1–S2 | NBRR2 | Creamy | Irregular | Smooth | Brown | 0.0 |
| 1–S3 | NBRR3 | White | Circular | Smooth | ND | 0.55 ± 1.15 |
| | NBRR4 | Gray | Circular | Rough | ND | 0.0 |
| | NBRR5 | Light gray | Circular | Rough | ND | 0.0 |
| | NBRR6 | White | Circular | Smooth | ND | 0.0 |
| 2–S1 | NBRM7 | Light gray | Circular | Rough | Red | 0.91 ± 0.5 |
| | NBRM8 | Gray | Irregular | Smooth | ND | 0.0 |
| | NBRM9 | White | Circular | Smooth | ND | 3.26 ± 0.66 |
| | NBRM10 | Gray | Punctiform | Smooth | Orange | 2.60 ± 0.32 |
| | NBRM11 | Gray | Punctiform | Rough | ND | 0.0 |
| 2–S2 | NBRM12 | Light orange | Irregular | Smooth | ND | 1.00 ± 0.47 |
| | NBRM13 | White | Irregular | Smooth | ND | 0.87 ± 0.37 |
| 2–S3 | NBRM14 | Medium gray | Circular | Smooth | ND | 0.0 |
| | NBRM15 | Creamy | Irregular | Rhizoid | Orange | 1.59 ± 0.32 |
| | NBRM16 | Creamy | Circular | Rhizoid | Brown | 1.03 ± 0.43 |
| | NBRM16 | Light orange | Circular | Smooth | Violet | 1.90 ± 1.13 |
| 3–S2 | NBRO18 | White | Circular | Rough | ND | 0.0 |
| | NBRO19 | Light gray | Circular | Rough | ND | 0.0 |
| | NBRO20 | Light gray | Circular | Rough | Blue | 0.0 |
| | NBRO21 | Purple | Circular | Smooth | Red | 0.0 |
| 4–S1 | NBRK22 | Medium gray | Punctiform | Smooth | Orange | 1.03 ± 0.73 |
| | NBRK23 | Medium gray | Punctiform | Smooth | Brown | 0.0 |
| | NBRK24 | White | Irregular | Rough | Red | 0.0 |
| 4–S2 | NBRK25 | Medium gray | Circular | Smooth | Red | 0.85 ± 0.41 |
| | NBRK26 | White | Irregular | Smooth | ND | 0.0 |
| | NBRK27 | White | Punctiform | Smooth | ND | 0.0 |
| | NBRK28 | White | Irregular | Smooth | Violet | 1.73 ± 0.95 |
| 4–S3 | NBRK29 | Light gray | Circular | Rough | ND | 0.0 |
| | NBRK30 | Light gray | Circular | Rough | Brown | 0.0 |
| | NBRK31 | Medium gray | Irregular | Smooth | ND | 0.0 |
| **Incidence of activity** | | | **Total isolates** | | **31** | **12** |
| | | | **%** | | **100** | **38.70** |

* The presented morphological features were recorded from the growth on isolation media (ISP–4).

*Average index of cellulase enzyme activity.

**Table S3.** Cultural characteristics of the actinomycete isolate NBRM9 allowed to grow on 7-ISP and other growth media.

| Media | Growth rate | Age (days) | Mycelium color | | Soluble pigments |
|---|---|---|---|---|---|
| | | | Aerial mycelium | Substrate mycelium | |
| ISP medium 1 (Liquid tryptone yeast extract) | Abundant | 7 | White (ISCC-NBS 263) | White (ISCC-NBS 263) | None |
| | | 14 | Pale yellow (ISCC-NBS 89) | Pale greenish yellow (ISCC-NBS 104) | None |
| | | 21 | Pale yellow (ISCC-NBS 89) | Pale orange yellow (ISCC-NBS 73) | None |
| ISP medium 2 (Yeast extract-malt extract agar) | Abundant | 7 | White (ISCC-NBS 263) | Pale yellow (ISCC-NBS 89) | None |
| | | 14 | White (ISCC-NBS 263) | Pale orange yellow (ISCC-NBS 73) | None |
| | | 21 | Yellowish white (ISCC-NBS 92) | Pale orange yellow (ISCC-NBS 73) | None |
| ISP medium 3 (Oatmeal agar) | No growth | 7-21 | - | - | - |
| ISP medium 4 (Inorganic salts-starch agar) | Abundant | 7 | White (ISCC-NBS 263) | White (ISCC-NBS 263) | None |
| | | 14 | Yellowish white (ISCC-NBS 92) | Yellowish gray (ISCC-NBS 93) | None |
| | | 21 | Yellowish white (ISCC-NBS 92) | Pale greenish yellow (ISCC-NBS 104) | None |
| ISP medium 5 (Glycerol asparagine agar) | Moderate | 7 | Pale yellow (ISCC-NBS 89) | Yellowish white (ISCC-NBS 92) | None |
| | | 14 | Pale yellow (ISCC-NBS 89) | Light yellow (ISCC-NBS 86) | None |
| | | 21 | Light orange yellow (ISCC-NBS 70) | Deep orange yellow (ISCC-NBS 69) | None |
| ISP medium 6 (Peptone yeast extract iron agar) | Moderate | 7 | Yellowish white (ISCC-NBS 92) | Vivid yellow (ISCC-NBS 82) | None |
| | | 14 | Yellowish white (ISCC-NBS 92) | Pale yellow (ISCC-NBS 89) | None |
| | | 21 | Yellowish gray (ISCC-NBS 93) | Strong orange yellow (ISCC-NBS 68) | None |
| ISP medium 7 (Tyrosine agar) | Moderate | 7 | White (ISCC-NBS 263) | Dark yellow (ISCC-NBS 88) | None |
| | | 14 | White (ISCC-NBS 263) | Dark yellow (ISCC-NBS 88) | None |
| | | 21 | White (ISCC-NBS 263) | Light yellowish brown (ISCC-NBS 76) | None |
| Starch-nitrate agar | Abundant | 7 | White (ISCC-NBS 263) | White (ISCC-NBS 263) | None |
| | | 14 | Pale greenish yellow (ISCC-NBS 104) | Light orange yellow (ISCC-NBS 70) | None |
| | | 21 | Pale orange yellow (ISCC-NBS 73) | Light orange yellow (ISCC-NBS 70) | None |

**Table S4.** Box–Behnken design levels of independent factors.

| No. | Factor | Variables | Units | Range | | |
|---|---|---|---|---|---|---|
| | | | | Minimum | Maximum | Mean |
| 1 | A | Temperature | °C | 30 | 50 | 40 |
| 2 | B | pH | - | 5 | 9 | 7 |
| 3 | C | Incubation time | Day | 3 | 7 | 5 |
| 4 | D | Substrate concentration | g | 1 | 5 | 3 |

**Table S5.** Box–Behnken design of optimization variables with experimental and predicted cellulase activity of *N. synnemataformans* NBRM9.

| Run No. | Variables | | | | Cellulase activity (U/mL) | |
|---|---|---|---|---|---|---|
| | A | B | C | D | Exp. response | Pred. response |
| 1 | 40 | 7 | 7 | 3 | 11.77 | 12.07 |
| 2 | 40 | 7 | 3 | 3 | 10.84 | 10.85 |
| 3 | 40 | 5 | 7 | 2 | 11.00 | 10.83 |
| 4 | 40 | 7 | 7 | 1 | 9.97 | 10.14 |
| 5 | 40 | 5 | 5 | 1 | 8.94 | 8.96 |
| 6 | 50 | 7 | 5 | 1 | 8.68 | 8.54 |
| 7 | 40 | 7 | 3 | 1 | 10.87 | 10.76 |
| 8 | 40 | 5 | 5 | 3 | 12.16 | 12.14 |
| 9 | 30 | 9 | 5 | 2 | 12.08 | 12.25 |
| 10 | 40 | 9 | 3 | 2 | 11.24 | 11.02 |
| 11 | 30 | 7 | 3 | 2 | 12.25 | 12.35 |
| 12 | 50 | 7 | 3 | 2 | 9.82 | 9.87 |
| 13 | 30 | 7 | 5 | 1 | 11.56 | 11.40 |
| 14 | 40 | 7 | 5 | 2 | 10.88 | 10.86 |
| 15 | 40 | 7 | 5 | 2 | 10.87 | 10.86 |
| 16 | 30 | 7 | 7 | 2 | 12.51 | 12.67 |
| 17 | 50 | 5 | 5 | 2 | 9.25 | 9.26 |
| 18 | 50 | 9 | 5 | 2 | 10.24 | 10.44 |
| 19 | 50 | 7 | 7 | 2 | 10.04 | 10.15 |
| 20 | 40 | 7 | 5 | 2 | 10.84 | 10.86 |
| 21 | 40 | 9 | 5 | 3 | 10.28 | 10.46 |
| 22 | 40 | 9 | 5 | 1 | 11.40 | 11.62 |
| 23 | 30 | 5 | 5 | 2 | 12.45 | 12.44 |
| 24 | 50 | 7 | 5 | 3 | 10.13 | 9.91 |
| 25 | 30 | 7 | 5 | 3 | 12.29 | 12.04 |
| 26 | 40 | 9 | 7 | 2 | 13.20 | 12.63 |
| 27 | 40 | 5 | 3 | 2 | 11.67 | 11.84 |

Exp. (experimental); Pred. (predicted).

**Table S6.** Estimated regression coefficients for the quadratic polynomial model and analysis of variance for the experimental results of cellulase production by *N. synnemataformans* NBRM9.

| Source | DF | Sum of squares | Mean of squares | F value | *p* value | Prob > F |
|---|---|---|---|---|---|---|
| Model | 14.00 | 33.6197 | 2.4014 | 31.56 | 0.000 | Significant |
| Linear | 4.00 | 22.7774 | 5.6944 | 74.83 | 0.000 | |
| A | 1.00 | 18.7143 | 18.7143 | 245.92 | 0.000 | |
| B | 1.00 | 0.7323 | 0.7323 | 9.62 | 0.009 | |
| C | 1.00 | 0.2737 | 0.2737 | 3.60 | 0.082 | |
| D | 1.00 | 3.0571 | 3.0571 | 40.17 | 0.000 | |
| Square | 4.00 | 3.0030 | 0.7507 | 9.87 | 0.001 | |
| $A^2$ | 1.00 | 0.0097 | 0.0097 | 0.13 | 0.727 | |
| $B^2$ | 1.00 | 0.4211 | 0.4211 | 5.53 | 0.037 | |
| $C^2$ | 1.00 | 1.0333 | 1.0333 | 13.58 | 0.003 | |
| $D^2$ | 1.00 | 0.6364 | 0.6364 | 8.36 | 0.014 | |
| 2-Way Interaction | 6.00 | 7.8393 | 1.3066 | 17.17 | 0.000 | |
| AB | 1.00 | 0.4670 | 0.4670 | 6.14 | 0.029 | |
| AC | 1.00 | 0.0004 | 0.0004 | 0.00 | 0.946 | |
| AD | 1.00 | 0.1303 | 0.1303 | 1.71 | 0.215 | |
| BC | 1.00 | 1.7134 | 1.7134 | 22.51 | 0.000 | |
| BD | 1.00 | 4.6897 | 4.6897 | 61.62 | 0.000 | |
| CD | 1.00 | 0.8387 | 0.8387 | 11.02 | 0.006 | |
| Error | 12.00 | 0.9132 | 0.0761 | | | |
| Lack-of-Fit | 10.00 | 0.9124 | 0.0912 | 227.29 | 0.004 | |
| Pure Error | 2.00 | 0.0008 | 0.0004 | | | |
| Total | 26.00 | 34.5330 | | | | Not significant |

$R^2$ (0.9736), adjusted $R^2$ (0.9427), predicted $R^2$ (0.8478), df (degree of freedom), highly significant ($p \leq 0.0001$), significant ($p \leq 0.05$), and nonsignificant ($p > 0.05$).

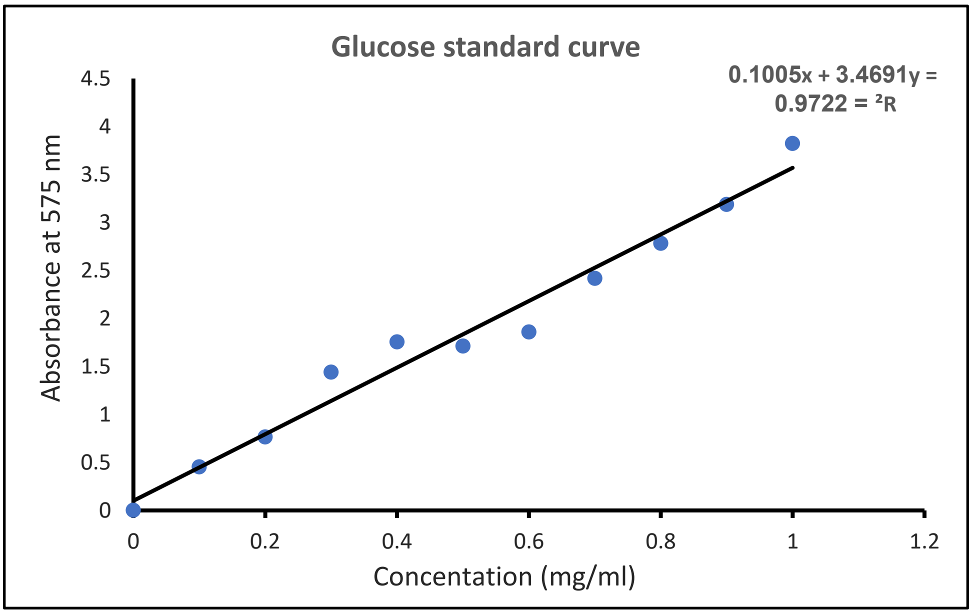


**Figure S1.** Glucose calibration curve.

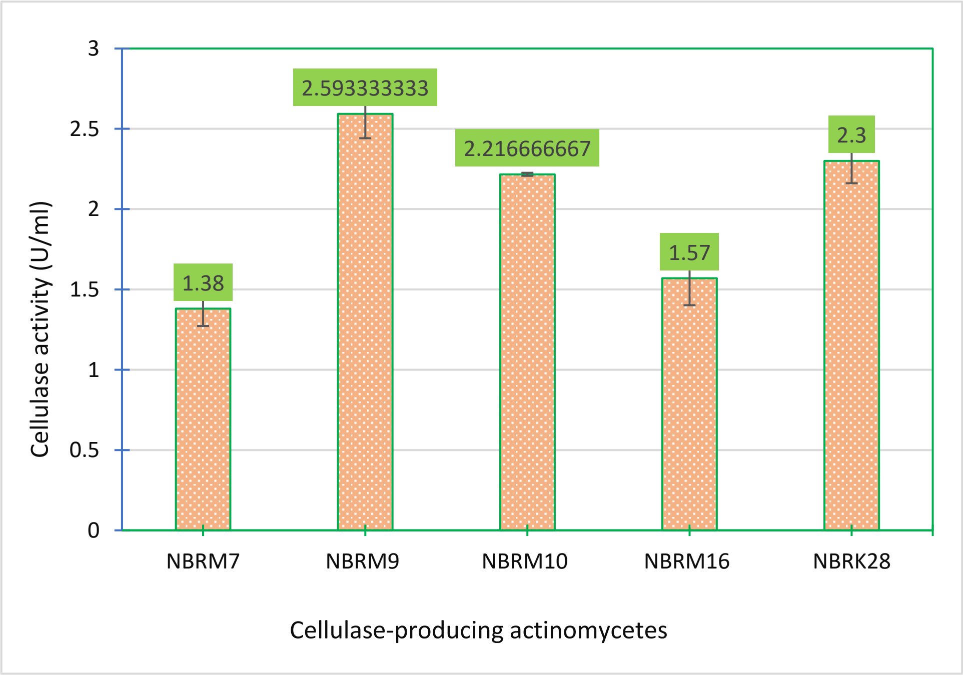


**Figure S2.** Cellulase activity of actinomycetes using CMC as the sole carbon source in liquid media.

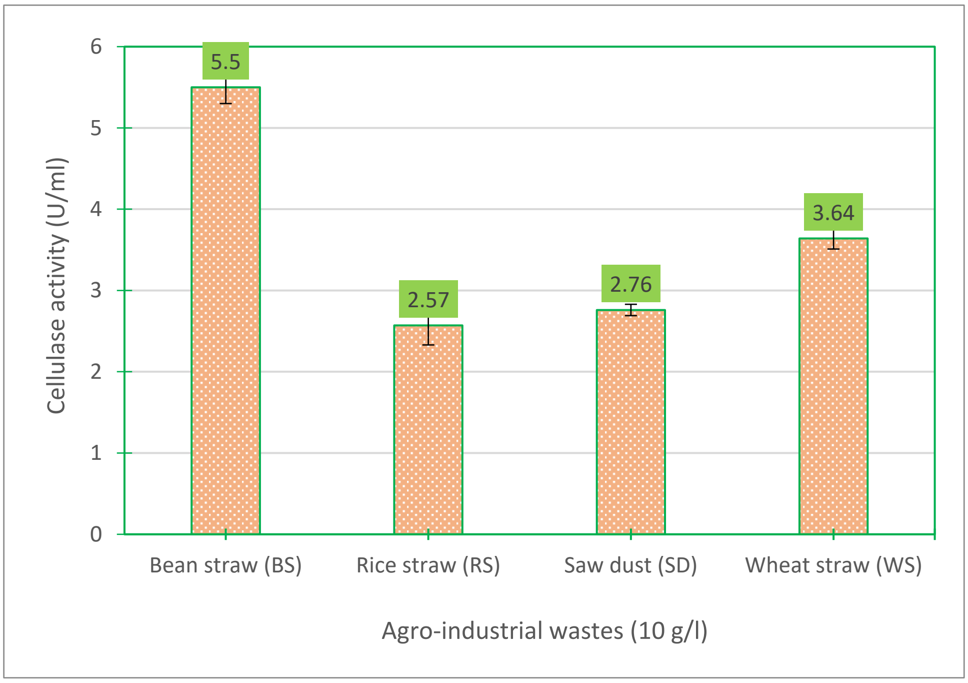


**Figure S3.** Cellulase production activities of the most effective actinomycete NBRM9 using the different agro-industrial wastes under SmF.

AIMS Press

© 2024 the Author(s), licensee AIMS Press. This is an open access article distributed under the terms of the Creative Commons Attribution License (http://creativecommons.org/licenses/by/4.0)
